# Effectiveness of a pharmacist-driven intervention in COPD (EPIC): study protocol for a randomized controlled trial

**DOI:** 10.1186/s13063-016-1623-7

**Published:** 2016-10-13

**Authors:** Erin Davis, Carlo Marra, John-Michael Gamble, Jamie Farrell, Joe Lockyer, J. Mark FitzGerald, Waseem Abu-Ashour, Charlie Gillis, John Hawboldt

**Affiliations:** 1 Memorial University School of Pharmacy, 300 Prince Philip Dr., St. John’s, NL A1B 3V6 Canada; 2 Memorial University Faculty of Medicine, Discipline of Family Medicine, 300 Prince Philip Dr., St. John’s, NL A1B 3V6 Canada; 3 Memorial University Faculty of Medicine, Discipline of Medicine, 300 Prince Philip Dr., St. John’s, NL A1B 3V6 Canada; 4 NL Eastern Health, Respiratory Medicine, 300 Prince Philip Dr., St. John’s, NL A1B 3V6 Canada; 5 Division of Respiratory Medicine, The University of British Columbia, 2775 Laurel Street, Vancouver, BC V5Z 1M9 Canada

**Keywords:** Chronic obstructive pulmonary disease, Cluster randomized controlled trial, Community pharmacy, Medication adherence, Pharmacist, Pharmacy practice research

## Abstract

**Background:**

Patients with chronic obstructive pulmonary disease (COPD) are often nonadherent with medications and have poor inhaler technique. Community pharmacists can help to improve health-related quality of life and overall outcomes in patients with COPD. We aim to measure the effectiveness of a systematic, pharmacist-driven intervention on patients with diagnosed COPD.

**Methods/design:**

This pragmatic, parallel-group, cluster randomized controlled trial is designed to determine the effectiveness of a multifactorial, pharmacist-led intervention on medication adherence, inhaler technique, health-related quality of life, health care resource utilization including COPD exacerbations, and use of medications. Participating pharmacies in Newfoundland and Labrador (NL), Canada will be randomly assigned to either the intervention or the control group. The intervention group will deliver an enhanced form of care that emphasizes COPD management. The control group will provide usual care and a COPD education pamphlet. Included patients will be aged 40 years or older, have a physician-confirmed diagnosis of COPD, and be able to answer questionnaires in English. The primary outcomes are the between-group difference in the change from baseline to 6 months in medication adherence using the Medication Possession Ratio (MPR) and the Morisky Medication Adherence Scale (MMAS-8). The secondary outcomes are also measured from baseline to 6 months, and include the proportion of patients with a clinically significant change in adherence, the proportion of patients defined as having “good adherence,” the mean MPR between groups, quality of life as measured by the St. George’s Respiratory Questionnaire, medication inhalation technique using a pharmacist-scored checklist, health care resource utilization and antibiotic and orally administered corticosteroid use for COPD exacerbations. Differences between groups will be analyzed at the individual patient level while controlling for clustering effect.

**Discussion:**

A pharmacist-led COPD intervention has the potential to improve patient medication adherence, thus increasing quality of life, possibly decreasing pulmonary exacerbations and reducing utilization of acute health care resources. Methods and results taken from this study could be used to enhance the delivery of COPD care by community pharmacists in a real-world setting. This would serve to enhance COPD population health and quality of life.

**Trial registration:**

International Standard Randomized Controlled Trial Number (ISRCTN) ISRCTN78138190, registered on 3 February 2016.

**Electronic supplementary material:**

The online version of this article (doi:10.1186/s13063-016-1623-7) contains supplementary material, which is available to authorized users.

## Background

### Background and rationale

Chronic obstructive pulmonary disease (COPD) is a respiratory disease characterized by a state of chronic inflammation, usually as a result of environmental toxins, leading to a progressive loss of airway function and systemic comorbidities [1, 2]. COPD is a significant cause of morbidity and mortality and also represents a high economic and social burden [3]. It is listed as the fifth leading cause of death in the world [4], and the fourth leading cause of death in Canada [5]. The overall societal cost of COPD in Canada in 2011, including direct and indirect costs of the disease, was CAN $4.52 billion [6]. Moreover, COPD exacerbations account for more than 50 % of the total health system costs of COPD [2, 7]. The prevalence of COPD is increasing, and although a large proportion of patients remain undiagnosed, they contribute a comparable health care burden to those who have been diagnosed [8].

The approach to COPD management is multifactorial and consists of nonpharmacological as well as pharmacological strategies in order to reduce symptoms, improve quality of life, reduce exacerbations, and slow disease progression [1, 2, 9, 10]. Unfortunately, rates of adherence for medication use in those with COPD are particularly low. The World Health Organization estimates a 50 % adherence rate for patients with COPD [11], while many studies report adherence rates in clinical practice to be between 40 and 60 % [12–17]. There are factors unique to COPD that predispose patients to adherence issues, including the chronic nature of the disease, complex medication regimens, significant comorbidities, and periods of disease stability between exacerbations [16, 18].

Nonadherence has a significant impact on a patient’s outcome, increasing hospitalization and exacerbation rates [10, 19, 20]. Indeed, multiple studies have shown an association between nonadherence in COPD and clinical and economic outcomes [21]. A post-hoc analysis of the Towards a Revolution in COPD (TORCH) trial indicated that patients with more than 80 % adherence had a mortality rate of 11.3 % as compared to 26.4 % in those with adherence of 80 % or below with annual hospitalization rates for exacerbations being 0.15 for adherent patients and 0.27 for nonadherent patients [10]. Furthermore, another study indicated that better adherence was associated with a 20 % reduction in annual hospitalizations [20].

Pharmacists, as readily accessible primary health care professionals with frequent interactions with patients, play a unique role in the health care system. Moreover, community pharmacies can act as cost-effective primary care platforms for improving medication adherence, inhalation technique, and health-related quality of life in COPD [22–25]. A multifactorial, individualized approach to COPD treatment has been suggested by many studies [2, 15, 16, 22–25], with pharmacists playing a major role.

Our multifactorial intervention represents the highest level of care available to COPD patients within the current scope of practice of pharmacists in Newfoundland and Labrador (NL), Canada, and allows us to compare the best possible care to the current level of care provided to COPD patients in the community.

### Objectives

The primary objective of this study is to test the effect of a multifactorial intervention at the community pharmacy level on patient adherence to respiratory medications in patients with COPD. Secondary objectives include assessing the impact of the intervention on quality of life, inhaler technique, and the sustainability and cost-effectiveness of these enhanced services. We hypothesize that the intervention will lead to improved adherence and more effective use of medication such as: better inhalation technique, and being prescribed more appropriate therapy for disease severity.

### Trial design

This trial is a pragmatic, parallel-group, cluster randomized controlled trial (RCT). Pharmacies will be allocated in a 1:1 ratio, and the data analyzed using the individual as the unit of analysis according to a superiority framework. A completed Standard Protocol Items: Recommendations for Interventional Trials (SPIRIT) checklist for the trial is available (see Additional file 1).

## Methods/design

### Study setting

The following study will be implemented in community pharmacies throughout NL. A complete list of study sites as of September 2016 is available in Additional file 2.

### Eligibility criteria

Inclusion restrictions were minimized in order to retain the pragmatic nature of the study design. All community pharmacies in NL holding a valid pharmacy licence will be eligible to participate.

The patient inclusion criteria are as follows:

1. Physician-diagnosed COPD
2. Age 40 years or older at trial enrollment
3. The ability to answer questionnaires in English

Patients will not be eligible to participate in the study if they have:

1. Severe disease, defined as a known Forced Expiratory Volume in 1 s (FEV1)/Forced Vital Capacity (FVC) of below 30 %
2. A diagnosis of dementia or a prescription for cholinesterase inhibitors
3. A terminal illness
4. Physician-diagnosed asthma
5. Participation in another clinical trial
6. They do not provide consent

### Interventions

Staff pharmacists working at all participating pharmacies from both arms of the study will be offered training on the design of the study, including how to administer questionnaires, proper patient recruitment, and consenting of patients. The intervention group pharmacists will also receive additional training on how to administer the intervention to a patient as well as an overall “refresher” on COPD management. In the event that additional staff pharmacists at the recruited pharmacies are interested in participating in the study, they will be provided with the required training as needed, (see Additional files 3, 4, and 5 for training materials).

**Fig. 1:**
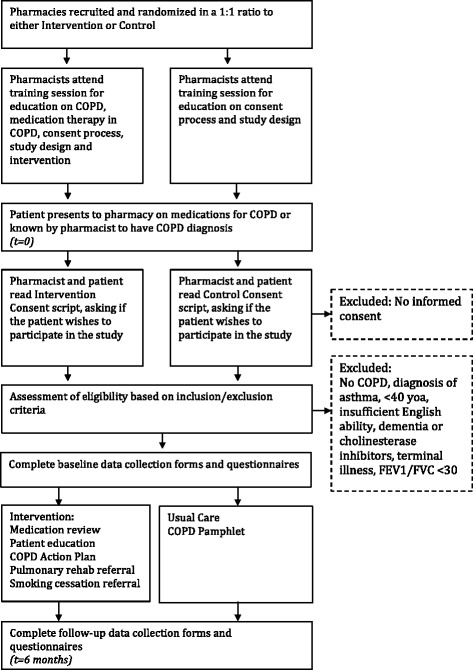
Study Flow Diagram

Interested patients will be identified through the use of inhaled medication or a known diagnosis of COPD. Posters and shelf-talkers will also be used to direct interested patients to the pharmacist for screening. The recruiting pharmacist will determine whether or not the patient meets the study criteria. The pharmacist will collect relevant information from the patient directly, or from their physician by telephone or fax in order to complete the Patient Screening Form. Figure 1 provides a flow chart of the consent and data collection process. Data collection and delivery of the intervention may be split into two pharmacy visits of no more than 2 weeks apart.

Our intervention will be administered to patients at their usual pharmacy after consent is obtained. The intervention involves six main strategies in addition to the COPD education pamphlet (see Additional file 6); (1) medication review, (2) patient education, (3) a written COPD Action plan provided in collaboration with their family physician (see Additional file 7), (4) patient referral to pulmonary rehabilitation in collaboration with their family physician, (5) provision of, or referral to, smoking cessation counseling (where applicable), and (6) referral to a community-based chronic disease self-management program.

1. Medication review
  Patients will have a thorough review of their current COPD medications. The review will consist of current medications, doses, dosage forms, duration and timelines of therapy, appropriateness of therapy, and patient expectations. Drug-related problems will be identified and recorded, and recommendations for their resolution will be forwarded to the patient’s primary health care provider
2. Patient education
  The education will consist of evaluating current inhaler technique and the subsequent correction or teaching where required. Pharmacists will also deliver adherence support strategies by determining knowledge deficits, understanding the patient’s expectations of their COPD therapy, and focusing on teaching about medications and administration techniques. The “teach-back technique” will be used [26–28]
3. COPD Action Plan
  A written “COPD Action plan” Form will be provided and explained to the patient. This action plan will inform the patient how to proceed when COPD symptoms worsen. This action plan will be developed in conjunction with the patient’s physician, where patients do not already have a standing prescription for antibiotics and oral steroids. The form is divided into two sections, each section having three subcategories. These include: (1) “My Symptoms” (I feel well, I feel worse, I feel much worse) and (2) “My Actions” (stay well, take action, call for help). This action plan is easy to read and simple to follow [29]. A copy of this will be provided to the patient and to the patient’s physician (faxed). When needed, a prescription suggestion for antibiotics and oral steroids will be provided to the physician for signature and fax back to the pharmacy, to facilitate the action plan
4. Pulmonary Rehabilitation
  In NL, access to specialized personnel or services is usually achieved through referral by the family practitioner. As such, all patients will have a request for referral to pulmonary rehabilitation sent to their family physician
5. Smoking cessation
  Pharmacists will also refer current smokers to smoking cessation services, or offer smoking cessation counseling within the pharmacy, where available
6. Community-based chronic disease self-management program
  Pharmacists will refer all patients to the community-based chronic disease self-management program, “Improving Health My Way.”
  Patients attending pharmacies assigned to the control group will receive a pamphlet on COPD and usual care according to the clinical judgment of the participating pharmacist. Care will not be limited or directed by the study team in any way. Patients will be able to withdraw from the study at any time. Research staff will be available to participating pharmacists to answer questions or provide support as necessary.

### Outcomes

The primary outcome is the difference in the change in the MPR from baseline to 6 months between the intervention and control groups. The World Health Organization (WHO) has defined adherence to long-term therapy as “the extent to which a person’s behavior (taking medication, following a diet, and/or executing lifestyle changes) corresponds with agreed recommendations from a health care provider” [30].

Medication adherence will be measured using both the Medication Possession Ratio (MPR) and the Morisky Medication Adherence Scale (MMAS-8) at baseline and after 6 months of follow-up. The MPR is the ratio of days of medication supplied over the 6-month follow-up [31], which will be calculated using prescription records collected from participating pharmacies. Prescription record data will be kept on all of the patient’s prescriptions. As in previous studies, a 10 to 15 % change in the MPR will be considered a minimal clinically important change [31, 32].

The MMAS-8 is a commonly used, validated, four-item, self-reported adherence measure that has been shown to be predictive of adherence to cardiovascular medications and blood pressure control [33, 34]. The minimal clinically important difference of the MMAS-8 is defined as a change of at least 2 points [35]. Secondary adherence outcomes include the proportion of patients with a clinically significant change in adherence, the proportion of patients defined as having “good adherence” and the mean MPR between groups. Similar to others, we will consider a threshold for good adherence to be an MPR of at least 80 % or a MMAS-8 score of 8 or greater [36, 37].

Secondary outcomes will be measured at baseline and 6 months and include: (1) quality of life, assessed by the St. George’s Respiratory Questionnaire, (2) medication inhalation technique using a pharmacist-scored scale, (3) health care resource utilization (frequency of physician visits, hospitalizations, emergency department visits, and pharmacy visits) as reported by the patient at 6 months, and (4) antibiotic and orally administered corticosteroid use for acute exacerbations of COPD (AECOPD) as reported by the patient at 6 months.

### Participant timeline

Patients will be recruited over a 12-month period between May 2016 and May 2017 and will be followed for 6 months. See Fig. 1 for an overview of the participant process.

### Sample size calculation

We based our sample size calculation on our primary outcome of change in adherence measured using the MPR. We assumed a baseline adherence of 50 % [16, 38], a minimal detectable difference of a 14 % absolute change, a standard deviation of 30 %, a correlation of 0.6 between baseline and follow-up measurement, a type 1 error rate of 5 %, a type 2 error rate of 20 %, and an intraclass correlation coefficient of 0.05 [39]. We corrected for correlation amongst patients within clusters using the inflation factor 1 + *p*(*m* − 1) where *m* is the mean number of observations per cluster and *p* is the intraclass correlation. We estimated that approximately 140 patients within at least 20 clusters would need to be enrolled to detect a 14 % change in adherence with 80 % power. We will maintain a cluster size of 20 pharmacies (10 intervention and 10 control) and aim to enroll seven patients per pharmacy, or 140 patients in total. The sample size for our study will, therefore, have 80 % power to detect a minimum difference of a 14 % change between the intervention and control groups.

### Recruitment

Pharmacists will be recruited primarily via email through the provincial pharmacy associations, with follow-up email and mail requests as needed. Patients presenting with a new prescription or refill for COPD medications will be approached by the pharmacist to gauge their interest in participating in the study.

We will provide ongoing support and work closely with pharmacies to ensure that recruitment targets are appropriate for the community they serve. Moreover, we will aim to monitor the recruitment process through regular communication via site visits, telephone calls, and emails to discuss any issues or challenges that might arise.

### Randomization

Randomization will be at the level of the community pharmacy. A random number list will be generated using Excel 2013 (Microsoft Corporation) and pharmacies will be assigned to either intervention or control in a 1:1 ratio by the research assistant as they are recruited.

### Blinding

It will not be possible for participating pharmacies to be blinded to which group they are assigned due to the nature of the intervention. The data analyst will be blinded to treatment assignment.

### Data collection methods

Pertinent demographic and contact information for participating pharmacies will be recorded. Patient study data will be collected using Data Collection Forms after pharmacists obtain their consent. Follow-up will last for 6 months after enrollment and the delivery of the intervention or usual care. All information will be stored at the pharmacy. The research assistant will collect the information in hardcopy or by fax for data entry. A blinded data analyst will conduct the final analysis.

Data collected will include: basic contact information (name, mailing address, email, and phone/cell number) as well as information related to the outcomes of the study, including questionnaires and prescription and health care resource utilization information. The Data Collection Forms are to be completed by the patient and pharmacist at baseline and 6 months. The research assistant will be available to assist in the completion of Data Collection Forms at the request of the pharmacist. Any harms reported by the participants will be recorded and included in the final manuscript.

### Data management

Patient information will be coded using unique numerical identifiers assigned by the research staff. All data entered electronically will be identified only by this code, and the master list will be kept in a locked cabinet in the locked office of the principal investigator. All electronic information will be kept in an encrypted file.

### Statistical methods

Baseline characteristics will be compared between the intervention and control groups at the pharmacy (cluster level) and patient level to assess for possible cluster imbalances. Differences in the primary and secondary outcomes of interest between the intervention and control groups will be measured at 6 months using the individual as the unit of analysis. Generalized linear mixed models will be used to account for the clustering effect at the pharmacy level. We will assume an exchangeable correlation structure, calculate robust standard errors, and specify the appropriate outcome distribution and link function for each model (e.g., binomial distribution and logit link function for dichotomous variables and a Gaussian distribution and identify link function for continuous variables). In addition to an unadjusted analysis, we will conduct a secondary analysis to adjust for potential differences in multiple baseline covariates at both the pharmacy level (e.g., pharmacy type, location, prescription volume, pharmacist to technician ratio) and the patient level (e.g., age, sex, baseline medication knowledge, baseline adherence, baseline inhaler technique). Standard model diagnostics will be conducted to check for model assumptions. All analysis will be intention-to-treat. Multiple imputation will be used to account for missing data.

### Protocol amendments

Any protocol amendments will be submitted to the NL Health Research Ethics Board for approval and noted in the registered protocol at the International Standard Randomized Controlled Trial Number (ISRCTN) register. Trial participants will be notified should relevant protocol changes be made.

### Access to data

All investigators, research assistants, and data analysts will have access to the trial data.

## Discussion

This study protocol presents the design of a pragmatic, cluster RCT to determine the effectiveness of a multifactorial pharmacist-driven intervention on medication adherence, inhaler technique, health-related quality of life, health care resource allocation, and use of medications including orally administered steroids and antibiotics during the study period.

COPD management in Canada remains suboptimal, with significant care gaps and patients experiencing poor outcomes and high exacerbation rates [40]. COPD has received considerably less attention in adherence research than asthma, diabetes, cardiovascular disease, and other chronic health conditions [41]. Thus, there is a need for additional evidence with regards to therapy adherence in COPD patients. Several adherence interventions have been studied in RCTs, but few of these have focused on COPD patients. Viswanathan et al, identified 62 RCTs in a systematic review, representing 18 different interventions designed to improve adherence to medications for chronic diseases [42]. However, none of the reviewed studies included COPD or addressed polypharmacy.

Chronic disease management would be assumed to be most successful when care is given through collaboration of health care team members and patients, and includes patient education and monitoring. Viswanathan et al. [42] concluded that the collaborative care approach was particularly effective in improving adherence. This has potential application for the treatment of COPD.

In a literature search of adherence interventions [43], key limitations in many studies addressing adherence revolve around three main points: reliance on inadequate adherence measures; inclusion of a convenient sample of patients; and assessments of intervention outcomes artificially boosted by attrition of least adherent participants. Our study overcomes these limitations; first by depending on pharmacy management software, giving insight into patient adherence; second by appropriate randomization to include a wide range of COPD patients through broad inclusion criteria and a follow-up plan; and third by assessing our primary outcome through both the MPR and the MMAS-8.

Additional strengths of our study include the pragmatic design of the study which allows the observed process to reflect real-world practice as accurately as possible. Systematic recruitment of participants via community pharmacies will increase the trial’s internal validity. Moreover, our choice of cluster randomization at the level of the pharmacy decreases the potential for contamination of the control as each pharmacist in either the intervention group or the control group will only be providing either usual care or the intervention, not both. Our intervention, though comprehensive, is relatively simple and easily applicable to the clinical setting and addresses both patient behavior and also the pharmacist-patient relationship. We will also measure outcomes beyond adherence alone, including disease-focused and patient-focused outcomes (e.g., health care resource utilization and quality of life).

There are also limitations of this study design. We are measuring our primary outcome indirectly via pharmacy records (MPR) and patient self-report (MMAS-8). Although the MMAS-8 tool has been validated, using direct adherence assessment tools, such as biochemical markers, respiratory device counters or electronic pillboxes, may potentially provide more accurate results. However, utilizing biochemical tests or potentially cumbersome respiratory device counters to measure adherence would be costly and impractical and would require repeat hospital visits and participant inconvenience, which would detract from the pragmatic nature of this study. There is also the potential for patients to interfere with respiratory device counters and thus they may not reliably provide superior measurement of adherence compared to the MPR and the MMAS-8. Another limitation includes potential selection bias due to pharmacists preferentially recruiting patients who they know are at higher risk of medication nonadherence to intervention pharmacies.

There will be no publication restrictions for the full trial results, and publication will be sought in peer-reviewed journals. The authors plan to hold stakeholder meetings to disseminate study results, as well as present the results at local and national conferences.

### Trial status

Pharmacy recruitment and intervention training began in February 2016 and patient recruitment began in May 2016.

## Additional files

Additional file 1: SPIRIT checklist. A completed SPIRIT checklist. (DOC 122 kb)
Additional file 2: List of study sites. A list of all of the pharmacies that have been enrolled in the trial to date. (PDF 30 kb)
Additional file 3: COPD refresher presentation. The training presentation used to refresh participating pharmacists’ knowledge of COPD and its treatment. (PDF 6378 kb)
Additional file 4: Intervention training presentation. The training presentation used to ensure all participating pharmacists are knowledgeable about and comfortable with the intervention used in the trial. (PDF 12947 kb)
Additional file 5: Study design presentation. The training presentation used to familiarize the participating pharmacists with the design and objectives of the trial. (PDF 18981 kb)
Additional file 6: COPD pamphlet. An educational pamphlet on COPD management from the Canadian Lung Association. (PDF 1041 kb)
Additional file 7: COPD Action Plan. An action plan to be developed by the pharmacists in the intervention arm, in conjunction with the patient and their primary prescriber as needed, to provide the patients with direction in the event of an acute exacerbation. This document was developed by the Canadian Thoracic Society and Lung Association. (PDF 405 kb)
