# Supplementary material for: Effectiveness of a pharmacist-driven intervention in COPD (EPIC): study protocol for a randomized controlled trial
Source: Trials. 2016 Oct 13;17:502. doi: 10.1186/s13063-016-1623-7 (PMC5064938; doi:10.1186/s13063-016-1623-7)
Supplement: Additional file 2: — List of study sites. A list of all of the pharmacies that have been enrolled in the trial to date. (PDF 30 kb) [file 13063_2016_1623_MOESM2_ESM.pdf]

Appendix A – Participating Pharmacies

ID#	Pharmacy	Allocation
1	Shoppers Drug Mart<br>P.O. Box 229 5 First Avenue<br>Pasadena NL A0L 1K0	Control
2	Shoppers Drug Mart<br>PO Box 807 30 Bond Street<br>Grand Falls-Windsor NL A2A 2M4	Intervention
3	The Drug Store Pharmacy<br>62 Prince Rupert Drive<br>Stephenville NL A2N3W7	Control
4	Sobey's Pharmacy<br>21 Cromer Ave.<br>Grand Falls-Windsor NL A2A 1X3	Control
5	Loblaw Pharmacy<br>166 Conception Bay Hwy.<br>Conception Bay South NL A1W 3A6	Intervention
6	Walmart Pharmacy<br>75 Kelsey Drive<br>St. John's NL A1B 5C8	Control
7	Lawton's Drugs<br>P.O. Box 1000 486 Main Street West<br>Lewisporte NL A0G 3A0	Control
8	Shoppers Drug Mart<br>Churchill Square 394 Elizabeth Avenue<br>St. John's NL A1B2X1	Intervention
9	Walmart Pharmacy<br>470 Topsail Road<br>St. John's NL A1E 2C3	Control
10	Breakwater Pharmacy<br>1604 Portugal Cove Road<br>Portugal Cove- St. Philips NL A1M 3G7	Intervention
11	Lawton's Drugs<br>645 Conception Bay Hwy, #102<br>Conception Bay South NL A1X 7L5	Intervention
12	Loblaw Pharmacy<br>240B Memorial Drive<br>Clarenville NL A5A 1N9	Control

**13**	Sinyard's Pharmacy<br>P.O. Box 359<br>Harbour Grace NL A0A2M0	Intervention
**14**	Baccalieu Trail Pharmacy<br>P. O. Box 250<br>Clarke's Beach NL A0A 1W0	Control
**15**	Lawton's Drugs<br>496 Topsail Rd<br>St. John's NL<br>A1E 2C2	Control
**16**	Walmart Pharmacy<br>42 Queen Street<br>Stephenville NL A2N 2Z5	Control
**17**	Sagona Drugs<br>P.O. Box 430 1 Canada Drive<br>Harbour Breton NL A0H 1P0	Intervention
**18**	Central Pharmacy<br>P.O. Box 496<br>Grand Falls-Windsor NL A2A 2J9	Intervention
**19**		
**20**		
