# Supplementary material for: Effectiveness of a pharmacist-driven intervention in COPD (EPIC): study protocol for a randomized controlled trial
Source: Trials. 2016 Oct 13;17:502. doi: 10.1186/s13063-016-1623-7 (PMC5064938; doi:10.1186/s13063-016-1623-7)

COPD THERAPEUTICS-INTERVENTION GROUP

Presenter Disclosure

- I have nothing to disclose

Learning Objectives

1. Identify patients at risk of COPD
2. Identify patients who may have undiagnosed COPD and would benefit from initiation of pharmacotherapy
3. Assess whether a patient with COPD is being optimally managed
4. Assess whether a patient is using the most appropriate inhaler device/delivery system
5. Promote non-pharmacological COPD management strategies
6. Utilize clinical services to aid COPD medication and device management

Outline

- COPD Background
  - What is COPD?
  - Pathophysiology
  - Symptoms
  - Risk Factors
  - Diagnosis
  - Classification
- Framework for Pharmacist's Role in COPD Management
  - Screening for Undiagnosed COPD
  - Drug Therapy Management
  - Assessment of Inhalation Technique
  - Clinical Services

Abbreviations

- **COPD** = Chronic Obstructive Pulmonary Disease
- **CTS** = Canadian Thoracic Society
- **FEV_1** = forced expiratory volume in one second
- **FVC** = forced vital capacity
- **GOLD** = Global Initiative for Chronic Obstructive Lung Disease
- **ICS** = inhaled corticosteroid
- **LAAC** = long-acting anticholinergic *or* **LAMA** = long-acting muscarinic antagonist
- **LABA** = long-acting $beta_2$-agonist
- **MRC** = Medical Research Council
- **PEV** = Peak expiratory volume
- **SAAC** = short-acting anticholinergic *or* **SAMA** = short-acting muscarinic antagonist
- **SABA** = short-acting $beta_2$-agonist
- **SABD** = short-acting bronchodilator

COPD BACKGROUND

What is COPD?

Definition

- "Respiratory disease largely caused by smoking, characterized by progressive, partially reversible airway obstruction and lung hyperinflation, systemic manifestations, and increasing frequency and severity of exacerbations"[1]

Epidemiology

- 3 million Canadians may have COPD (1.6 million may be undiagnosed)[2]
- 4th leading cause of death in Canada[3]
- Global prevalence is 9-10%[4]
- One of top 5 causes of death worldwide[5]
- Only chronic disease in which mortality is still increasing[6]

1. O'Donnell DE et al. Can Respir J. 2008;15(Suppl A):1A-8A. 2. Canadian Lung Association. http://www.lung.ca/pdf/copd/COPD_in_Canada.pdf. 3. Statistics Canada. http://www.statcan.gc.ca/tables-tableaux/sum-som/l01/cst01/hlth36a-eng.htm. 4. Bryant J et al. http://respiratory-research.com/content/14/1/109#B22. 5. World Health Organization. http://www.who.int/mediacentre/factsheets/fs310/en/. 6. Benady, S. http://www.lung.ca/cts-sct/pdf/COPDReport_E.pdf.

Pathophysiology[1]

Mechanism underlying airflow limitation in COPD[2]

- Small airways disease
- Airway inflammation
- Airway fibrosis
- Luminal plugs
- Increased airway resistance

- Lung tissue destruction
- Loss of alveolar attachments
- Decrease of elastic recoil

AIRFLOW LIMITATION

1. O'Donnell DE et al. Can Respir J. 2008;15(Suppl A):1A-8A. 2. Global Initiative for Chronic Obstructive Lung Disease (GOLD). http://www.goldcopd.org/uploads/users/files/GOLD_Report_2014_Jun11.pdf.

Symptoms

Chronic Cough[1,2]	Sputum Production[1,3]	Dyspnea[1,2]
• Often first symptom to develop<br>• [illegible]<br>• [illegible] intermittently but later occurs daily and throughout the day<br>• Seldom entirely nocturnal	• Small volumes produced after coughing bouts [illegible]<br>[illegible] exacerbations	• Cardinal symptom of COPD<br>• [illegible]<br>[illegible] persistent and progressive<br>• Described as increased effort to breath, heaviness or gasping[2]

PRACTICE PEARL
Assess all patients looking to purchase OTC products for a non-productive or phlegmy cough to determine the nature and pattern of their symptoms and their smoking status.

1. Black A. http://www.ajol.info/index.php/cme/article/download/50284/38971. 2. Global Initiative for Chronic Obstructive Lung Disease (GOLD). http://www.goldcopd.org/uploads/users/files/GOLD_Report_2013_Feb20.pdf. 3. Currie GP. ABC of COPD. 2011.

Dyspnea Scale

The MRC Dyspnea Scale[1,2]

Grade	Description
0	Not troubled by breathlessness except with strenuous exercise
[illegible]	[illegible]
[illegible]	[illegible] the level
3	Stops for breath after walking about 100 yards (90 m) or after a few minutes on the level
4	Too breathless to leave the house or breathless when dressing or undressing

PRACTICE PEARL
Utilize a modified version of the MRC Dyspnea Scale to assess the efficacy of current pharmacotherapy and disease impact.

1. O'Donnell DE et al. Can Respir J. 2008; 15(Suppl A): 1A-8A. 2. O'Donnell DE et al. Can Respir J. 2007;14(Suppl B):5B-32B.

Risk Factors[1,2,3,4]

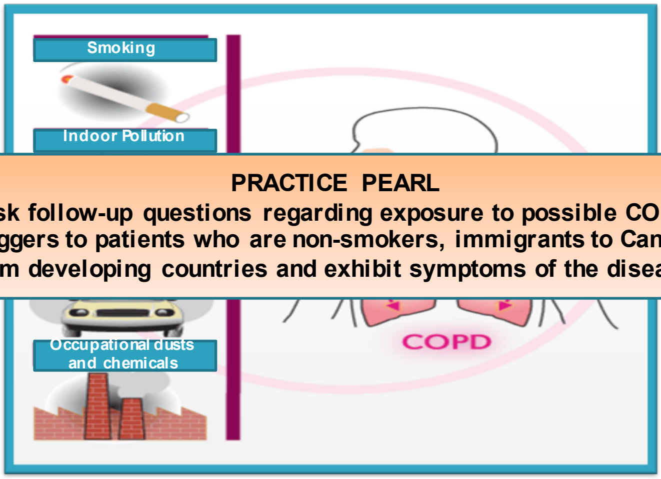

PRACTICE PEARL
Ask follow-up questions regarding exposure to possible COPD triggers to patients who are non-smokers, immigrants to Canada from developing countries and exhibit symptoms of the disease.

1. Currie GP. ABC of COPD. 2011. 2.. Public Health Agency of Canada. http://www.phac-aspc.gc.ca/cd-mc/crd-mrc/copd-mpoc-eng.php. 3. Global Initiative for Chronic Obstructive Lung Disease (GOLD). http://www.goldcopd.org/uploads/users/files/GOLD_Report_2014_Jun11.pdf. 4. Image from Doctor Tipster. http://www.doctortipster.com/3442-chronic-obstructive-pulmonary-disease-copd-risk-factors-diagnosis-clinical-forms-complications-evolution-and-prognostic.html.

Diagnosis[1,3]

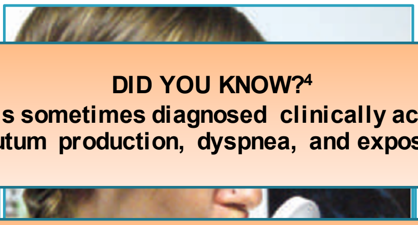

DID YOU KNOW?[4]
In practice, COPD is sometimes diagnosed clinically according to reported chronic cough, sputum production, dyspnea, and exposure to risk factors.

maximal inspiration

FVC maneuver

PRACTICE PEARL
Encourage patients to get tested!
Know the locations of walk-in clinics in your area if a patient does not have a family physician or if access may be delayed.

1. . McIvor RA. http://www.e-therapeutics.ca/tc.showChapter.action?documentId=c0040#c0040n00008. 2. Black A. http://www.ajol.info/index.php/cme/article/download/50284/38971. 3. O'Donnell DE et al. Can Respir J. 2007;14(Suppl B):5B-32B. 4. Johns R. Pharm Prac. 2013.

Classification[2,3]

Canadian Thoracic Society COPD classification of severity by symptoms and disability, and impairment of lung function[1]	
**Classification by symptoms and disability**	
**COPD stage**	Symptoms
**Mild**	Shortness of breath from COPD when hurrying on the level or walking up a slight hill
**Moderate**	Shortness of breath from COPD causing the patient to stop after walking approximately 100 m (or after a few minutes) on the level
**Severe**	Shortness of breath from COPD resulting in the patient being too breathless to leave the house, breathless when dressing or undressing, or the presence of chronic respiratory failure or clinical signs of right heart failure
**Classification by impairment of lung function**	
**COPD stage**	Spirometry (postbronchodilator)
**Mild**	$FEV_1 \geq 80\%$ predicted, $FEV_1/FVC < 0.7$
**Moderate**	$50\% \leq FEV_1 < 80\%$ predicted, $FEV_1/FVC < 0.7$
**Severe**	$30\% \leq FEV_1 < 50\%$ predicted, $FEV_1/FVC < 0.7$
**Very severe**	$FEV_1 < 30\%$ predicted, $FEV_1/FVC < 0.7$

1. O'Donnell DE et al. Can Respir J. 2007;14(Suppl B):5B-32B. 2. Black A. http://www.ajol.info/index.php/cme/article/download/50284/38971. 3. McIvor RA. http://www.e-therapeutics.ca/search#.

COPD Combined Assessment[1,2]

When assessing risk, choose the highest risk according to GOLD grade or exacerbation history. (One or more hospitalizations for COPD exacerbations should be considered *high risk*.)

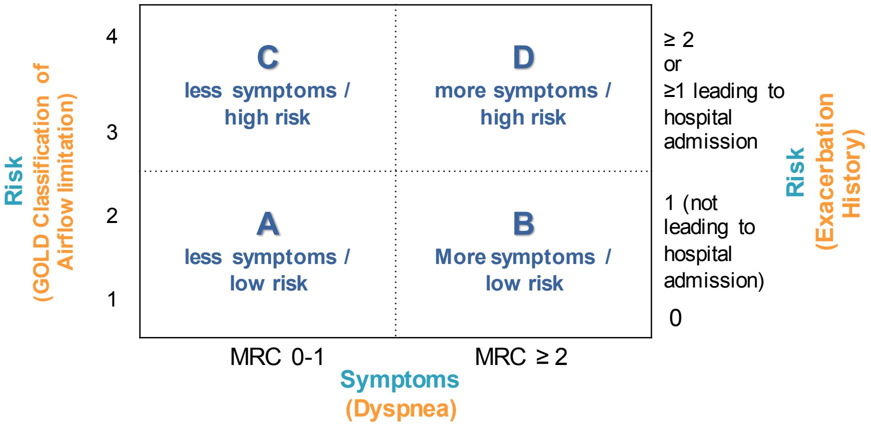

1. Adapted from Global Initiative for Chronic Obstructive Lung Disease (GOLD). http://www.goldcopd.org/uploads/users/files/GOLD_Pocket_2014_Jun11.pdf. 2. Adapted from Jackson LD. OPA Conference; 2014.

FRAMEWORK FOR PHARMACIST ROLE IN COPD MANAGEMENT

Goals of COPD Management[1,2]

- Alleviate dyspnea and other respiratory symptoms
- Improve exercise tolerance
- Improve health status

→ Reduce symptoms

- Prevent disease progression
- Reduce frequency and severity of exacerbations
- Treat exacerbations and complications of the disease
- Reduce mortality

→ Reduce risk

1. O'Donnell DE et al. Can Respir J. 2008;15(Suppl A):1A-8A. 2. Global Initiative for Chronic Obstructive Lung Disease (GOLD). http://www.goldcopd.org/uploads/users/files/GOLD_Pocket_2014_Jun11.pdf.

Comprehensive Approach to Management of COPD[1,2]

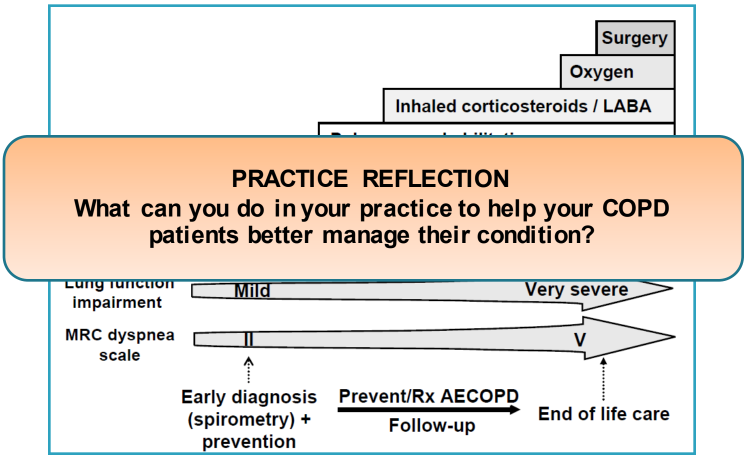

PRACTICE REFLECTION
What can you do in your practice to help your COPD patients better manage their condition?

1. O'Donnell DE et al. Can Respir J. 2008;15(Suppl A):1A-8A. 2. Vestbo J et al. Am J Respir Crit Care Med 2013;187:347-65.

Framework for Pharmacist Role

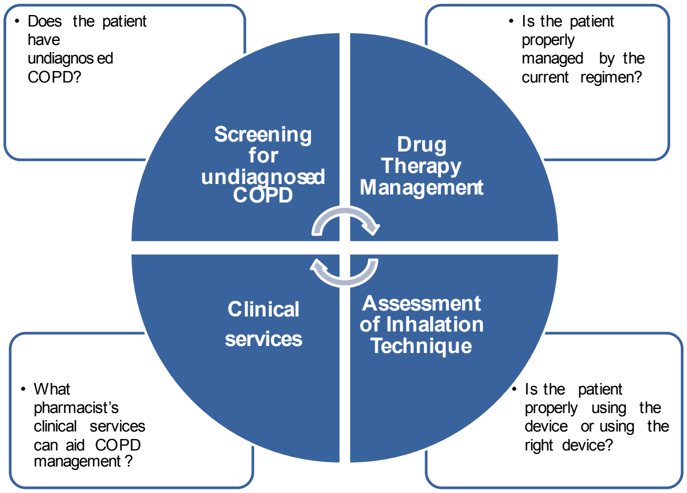

Framework for Pharmacist Role

1. **Does the patient have undiagnosed COPD?**
2. Is the patient properly managed by the current regimen?
3. Is the patient properly using the device or using the right device?
4. What pharmacist's services can aid COPD management?

Screening for Undiagnosed COPD[1,2,3]

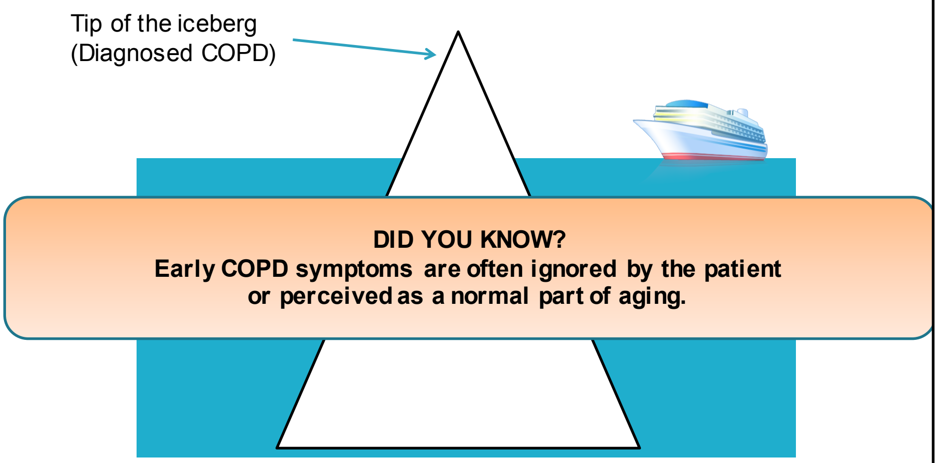

1. Doherty DE. J Am Med Dir Assoc. 2003;4(5 Suppl):S116-S120. 2. Currie GP. ABC of COPD. 2011. 3. O'Donnell DE et al. Can Respir J. 2008;15(Suppl A):1A-8A.

Screening for Undiagnosed COPD[1,2,3]

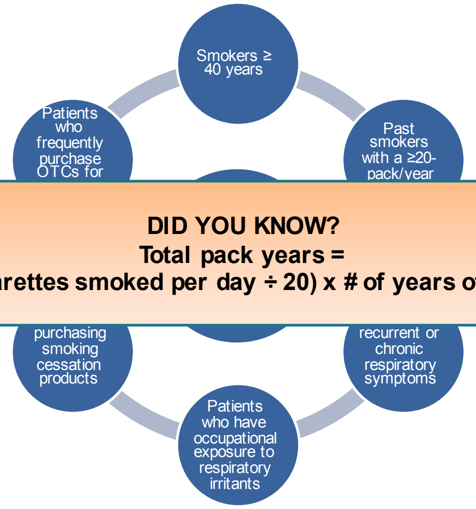

DID YOU KNOW?
Total pack years =
(# of cigarettes smoked per day ÷ 20) x # of years of smoking

1. O'Donnell DE et al. Can Respir J. 2008;15(Suppl A):1A-8A. 2. Ortiz G. http://www.advanceweb.com/web/AstraZeneca/focus_on_asthma_copd_issue5_Bronchodilator/issue5.html.

Screening Tools

Canadian Lung Health Test[1]			
**If you are over 40 and smoke or used to smoke, you may already have COPD. Take this quick test to screen for symptoms of COPD:**			
		Yes	No
**1.**	Do you cough regularly?		
**2.**	Do you cough up phlegm regularly?		
**3.**	Do even simple chores make you short of breath?		□
**4.**	Do you wheeze when you exert yourself (exercise, go upstairs)?	□	□
**5.**	Do you get many colds, and do your colds usually last longer than your friends' colds?	□	□

B.R.E.A.T.H.S.

- **Breathlessness**
- **Repeat infections**
- **Exacerbations**
- **Activity limitation**
- **Tiredness**
- **Heavy chest (cough and/or chest tightness)**
- **Sputum production**

1. Canadian Lung Association. [illegible]

Meet Teresa

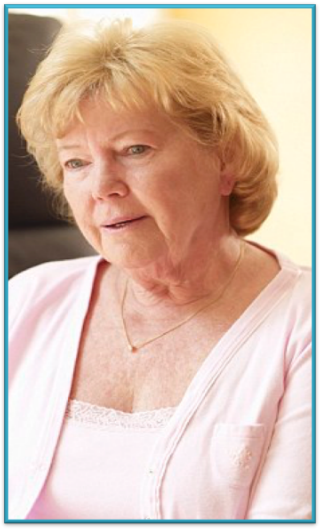

- Regular pharmacy patient
- 61-year old widow
- 3 week phlegmy cough
- 2 colds this year
- Describes feeling a little short of breath even when not sick
- Current non-smoker with 15-year pack history of smoking
- Current medications:
  - Candesartan 16mg od
  - Hydrochlorothiazide 25 mg od
  - Atorvastatin 40mg od
  - Zopiclone 7.5 mg hs prn
- Looking for an OTC product for daily cough

Does Theresa have COPD?

Does Theresa have COPD?

✔ Chronic cough

✔ Sputum production

[illegible]

PHARMACIST INTERVENTION
Advised Theresa to return to her physician for referral for COPD testing. Contacted physician to inform him of recommendation and patient's screening results.

Lung Health test

- ✔ Do you cough regularly?
- ✔ Do you cough up phlegm regularly?
- ✔ Do even simple chores make you short of breath?
- ✔ Do you wheeze when you exert yourself (exercise, go upstairs)?
- ✔ Do you get many colds, and do your colds usually last longer than your friends' colds?

Framework for Pharmacist Role

1. Does the patient have undiagnosed COPD?
2. **Is the patient properly managed by the current regimen?**
3. Is the patient properly using the device or using the right device?
4. What pharmacist's services can aid COPD management?

COPD Inhaled Pharmacotherapy[1,2]

SABA	SAMA (SAAC)	SAMA+ SABA	LABA	LAMA (LAAC)	LAMA+ LABA	ICS+ LABA
Salbutamol sulfate	Ipratropium bromide	Ipratropium bromide + Salbutamol sulfate	Salmeterol xinafoate	Tiotropium bromide	Glycopyrronium bromide + Indacaterol maleate	Budesonide + Formoterol fumarate dihydrate
Terbutaline sulfate		Ipratropium bromide + Fenoterol hydrobromide	Formoterol fumarate	Glycopyrronium bromide	Umeclidinium bromide + Vilanterol trifenatate	Fluticasone propionate + Salmeterol xinafoate
			Formoterol fumarate dihydrate	Aclidinium bromide	Tiotropium bromide monohydrate + Olodaterol hydrochloride	Fluticasone furoate + Vilanterol trifenatate
			Indacaterol maleate	Umeclidinium bromide	Aclidinium bromide + Formoterol fumarate dihydrate	

1. O'Donnell DE et al. Can Respir J. 2008;15(Suppl A):1A-8A. 2. McIvor RA. http://www.e-therapeutics.ca/search#.

COPD Inhaled Pharmacotherapy

- **SABAs**[1,2]
  - Salbutamol and Terbutaline
  - Recommended prn in all stages of disease severity for immediate symptom relief
  - Sometimes prescribed as initial therapy in patients with symptoms that are only noticeable with exertion and who have relatively little disability
- **SAMAs**[1,2]
  - Ipratropium
  - Available as a metered-dose inhaler or solution for nebulization
  - Slower onset of action than SABAs but a longer duration of action (up to 8 hours)
  - Less effective than tiotropium
  - Sometimes prescribed as initial therapy in patients with symptoms that are only noticeable with exertion and who have relatively little disability

1. O'Donnell DE et al. Can Respir J. 2008;15(Suppl A):1A-8A. 2. McIvor RA. http://www.e-therapeutics.ca/search#.

COPD Inhaled Pharmacotherapy

- **SAMAs + SABAs**[1,2,3]
  - Combination products result in greater improvement in lung function with lower or similar incidence of adverse effects
  - Ipratropium + Salbutamol
    - Indicated for treatment of bronchospasm associated with COPD
    - Available as solution for nebulization and soft mist inhaler
  - Ipratropium + Fenoterol
    - Indicated for the treatment of bronchospasm associated with acute severe exacerbations of COPD (and bronchial asthma)
    - Available as a solution for nebulization

1. McIvor RA. http://www.e-therapeutics.ca/search#. 2. O'Donnell DE et al. Can Respir J. 2008;15(Suppl A):1A-8A. Boehringer Ingelheim (Canada) Ltd. http://www.boehringer-ingelheim.ca/content/dam/internet/opu/ca_EN/documents/humanhealth/product_monograph/DuoventPMEN.pdf.

COPD Inhaled Pharmacotherapy

- **LABAs**[1,2,3]
  - More sustained improvements in pulmonary function, chronic dyspnea and health status than SABAs in patients with moderate to severe COPD
  - Salmeterol = long-acting, slow onset, dry powder inhaled
  - Formoterol = long-acting, rapid onset, dry powder inhaled
  - Indacaterol = once daily, ultra-long-acting, rapid onset, dry powder inhaled
- **LAMAs**[1,2,3,4]
  - More sustained effects in pulmonary function, chronic activity-related dyspnea and health status compared with regular dose ipratropium or placebo in patients with moderate to severe COPD
  - Tiotropium = once daily, long-acting dry powder and soft mist inhaled
  - Glycopyrronium = once daily, long-acting, rapid onset, dry powder inhaled
  - Aclidinium = twice daily, long-acting, dry powder inhaled
  - Umeclidinium = once daily, long-acting, dry powder inhaled

1. McIvor RA. http://www.e-therapeutics.ca/search#. 2. O'Donnell DE et al. Can Respir J. 2008;15(Suppl A):1A-8A 3. Johns R. Pharm Prac. 2013. 4. Jackson LD. OPA Conference; 2014. 5. Australian Prescriber. http://www.australianprescriber.com/magazine/38/2/drug/1086.pdf.

COPD Inhaled Pharmacotherapy

- **LAMAs + LABAs**[1]
  - Maximizes bronchodilation and lung deflation
  - Recommended in moderate to severe disease with persistent symptoms and infrequent exacerbations (<1 per year for at least 2 consecutive years)
  - Glycopyrronium + Indacaterol
  - Umeclidinium + Vilanterol
  - Tiotropium + Olodaterol
  - Aclidinium + Formoterol fumarate dihydrate
- **ICSs + LABAs**[1,2]
  - Not recommended as monotherapy
  - More effective than either drug alone in terms of exercise endurance, symptom control, lung function and exacerbation rates
  - Recommended to add to LAAC for patients with moderate to severe COPD and repeated exacerbations (≥1 per year for at least 2 consecutive years)
  - Budesonide + Formoterol fumarate dihydrate
  - Fluticasone + Salmeterol
  - Fluticasone + Vilanterol

1. McIvor RA. http://www.e-therapeutics.ca/search#. 2. O'Donnell DE et al. Can Respir J. 2008;15(Suppl A):1A-8A.

COPD Pharmacotherapy

- **PDE-4 Inhibitor**[1]
  - Roflumilast
  - Add-on therapy to bronchodilators for the maintenance treatment of severe COPD associated with chronic bronchitis in adult patients with a history of frequent exacerbations
  - Data support addition to foundation bronchodilatory therapy to reduce exacerbations, either before or in addition to introduction of ICS/LABA
- **Oral Systemic Corticosteroids**[1]
  - Improve lung function and shorten length of hospital stay and reduce risk of early relapse
  - For AECOPDs: 30–40 mg once daily po × 5–14 days
  - Tapering not necessary if course < 2 weeks
  - No role in COPD maintenance therapy

1. McIvor RA. http://www.e-therapeutics.ca/search#.

CTS Recommendations for Optimal Pharmacotherapy[1]

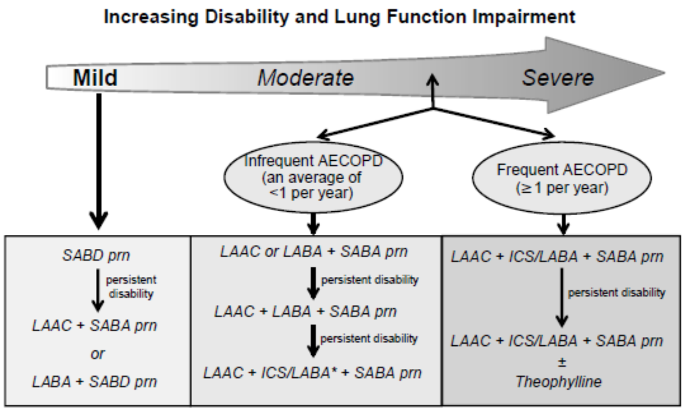

1. O'Donnell DE et al. Can Respir J. 2008;15(Suppl A):1A-8A. 2. Johns R. Pharm Prac. 2013.

GOLD Recommendations for Pharmacological Management of COPD[1]

Patient Group	Recommended First Choice	Alternative Choice	Other Possible Treatments
A	SAAC prn or SABA prn	LAAC or LABA or SABA + SAAC	Theophylline
B	LAAC or LABA	LAAC and LABA	SABA and/or SAAC Theophylline
C	ICS + LABA or LAAC	LAAC and LABA or LAAC and PDE-4 Inhibitor or LABA and PDE-4 Inhibitor	SABA and/or SAAC Theophylline
D	ICS + LABA and/or LAAC	ICS + LABA and LAAC or ICS + LABA and PDE-4 Inhibitor or LAAC and LABA or LAAC and PDE-4 Inhibitor	Carbocysteine SABA and/or SAAC Theophylline

1. Adapted from Global Initiative for Chronic Obstructive Lung Disease (GOLD). http://www.goldcopd.org/uploads/users/files/GOLD_Pocket_2014_Jun11.pdf.

Assessment of Adherence[1]

Intentional[2]

- Deliberate discontinuation or reduction in use of therapy during periods of symptom remission
- Causes:
  - [illegible] erroneous understanding of goals of treatment

Unintentional[2]

- Non-adherence to treatment advice due to reasons out of patient's control
- Causes:
  - [illegible]
  - Musculoskeletal problems
- Complex medication regimen
  - Polypharmacy
  - Multiple devices

PRACTICE PEARL
Explore ways to simplify medication regimen by recommending the prescribing of once daily agents to physicians if appropriate and ensuring the patient is using one type of inhalation device if possible.

1. Bourbeau J et al. Thorax. 2008;63(9):831–838. 2. Bryant J et al. http://respiratory-research.com/content/14/1/109#B22.

Assessment of Adherence

Over-utilizing medications during periods of respiratory distress

Non-adherence

PRACTICE PEARL[2]
Discuss drug information with patients to set appropriate expectations. Allow the patient to talk about their perceptions of the benefits and costs of the drug and provide patients an opportunity to identify and discuss their concerns and perceived barriers.

Underuse of medications is most common

Adherence to inhaled and oral medications **41.3% - 57.0%**

Lack of physical activity

1. Bryant J et al. http://respiratory-research.com/content/14/1/109#B22. 2. Bourbeau J et al. Thorax. 2008;63(9):831–838.

Assessment of Dyspnea

Modified Medical Research Council Questionnaire for Assessing the Severity of Breathlessness[1]			
**Grade**	**Severity**	**Description of Breathlessness Related to Activities**	
**0**	N/A	I only get breathless with strenuous exercise.<br>• Pharmacist: Do you only get short of breath when you take in strenuous exercise such as running, climbing stairs, or dancing?	◻
**1**	Mild	I get short of breath when hurrying on level ground or walking up a slight hill. [illegible]	◻
[illegible]	[illegible]	[illegible] breath? Do you have to stop for breath when you walk at your own pace?	[illegible]
**3**	Moderate	I stop for breath after walking about 100 yards (90 m) or after a few minutes on level ground.<br>• Pharmacist: Do you get short of breath when walking on a level surface for a few minutes?	◻
**4**	Severe	I am too breathless to leave the house or I am breathless when dressing.<br>• Pharmacist: Are you so short of breath that you have trouble leaving the house or become short of breath when dressing?	◻

PRACTICE PEARL
Ask questions that are indicative of the different COPD grades to assess severity of dyspnea, monitor progress and/or response to therapy.

1. Adapted from Global Initiative for Chronic Obstructive Lung Disease (GOLD). http://www.goldcopd.org/uploads/users/files/GOLD_Pocket_2014_Jun11.pdf.

Assessment of AECOPDs

PRACTICE PEARL[2]
Consider referring to AECOPDs as "COPD lung attacks" when speaking with patients that may not appreciate the seriousness of COPD.

increase in the use of maintenance medications

are thought to be infectious in nature

AECOPDs now account

PRACTICE PEARL[1]
Assess all patients presenting with a prescription for a respiratory infection to assess whether they have been diagnosed with COPD or are smoker or ex-smokers. Focus on the production and colour of phlegm.

readmission rate than other chronic illnesses

1. O'Donnell DE et al. Can Respir J. 2007;14(Suppl B):5B-32B. 2. Benady, S. http://www.lung.ca/cts-sct/pdf/COPDReport_E.pdf.

COPD Action Plans

- What is an action plan?
  - A COPD Action Plan is a written contract between the patient and their health care team. It will tell them how to manage their COPD flare-ups.
  - It is used along with any other information they get from their health care team about managing their COPD every day.
  - The Action Plan will help the patient and their caregivers to quickly recognize and act to treat the patient's flare-ups.

1. Canadian Thoracic Society. http://www.respiratoryguidelines.ca/sites/all/files/CTS_COPD_updated_Action_Plan_editable_PDF.pdf. 2. Kaplan, A. : http://www.cfp.ca/content/55/1/58.full.pdf+html.

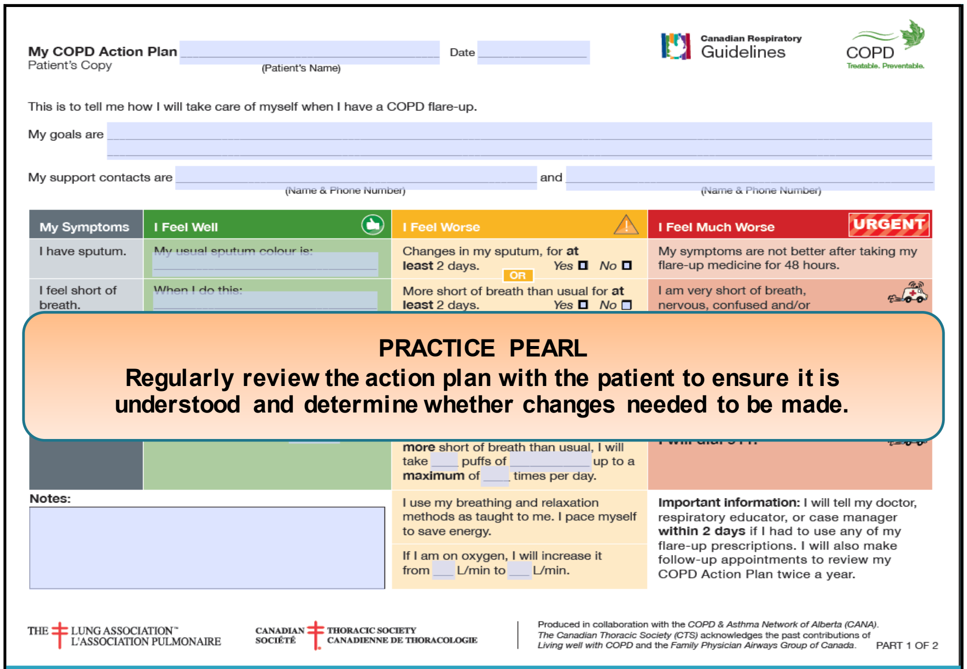

My COPD Action Plan ______ Date ______
Patient's Copy (Patient's Name)

Canadian Respiratory Guidelines
COPD Treatable. Preventable.

This is to tell me how I will take care of myself when I have a COPD flare-up.

My goals are ______

My support contacts are ______ (Name & Phone Number) and ______ (Name & Phone Number)

My Symptoms	I Feel Well	I Feel Worse	I Feel Much Worse URGENT
I have sputum.	My usual sputum colour is:	Changes in my sputum, for **at least** 2 days. Yes ☐ No ☐ OR	My symptoms are not better after taking my flare-up medicine for 48 hours.
I feel short of breath.	When I do this:	More short of breath than usual for **at least** 2 days. Yes ☐ No ☐	I am very short of breath, nervous, confused and/or
		**more** short of breath than usual, I will take ___ puffs of ______ up to a **maximum** of ___ times per day.	I will dial 911.
Notes:		I use my breathing and relaxation methods as taught to me. I pace myself to save energy.	**Important information:** I will tell my doctor, respiratory educator, or case manager **within 2 days** if I had to use any of my flare-up prescriptions. I will also make follow-up appointments to review my COPD Action Plan twice a year.
		If I am on oxygen, I will increase it from ___ L/min to ___ L/min.	

THE LUNG ASSOCIATION™ L'ASSOCIATION PULMONAIRE
CANADIAN THORACIC SOCIETY SOCIÉTÉ CANADIENNE DE THORACOLOGIE

Produced in collaboration with the *COPD & Asthma Network of Alberta (CANA)*. *The Canadian Thoracic Society (CTS)* acknowledges the past contributions of *Living well with COPD* and the Family Physician Airways Group of Canada. PART 1 OF 2

PRACTICE PEARL
Regularly review the action plan with the patient to ensure it is understood and determine whether changes needed to be made.

1. Canadian Thoracic Society. http://www.respiratoryguidelines.ca/sites/all/files/CTS_COPD_updated_Action_Plan_editable_PDF.pdf. 2. Kaplan, A. : http://www.cfp.ca/content/55/1/58.full.pdf+html.

Teresa again

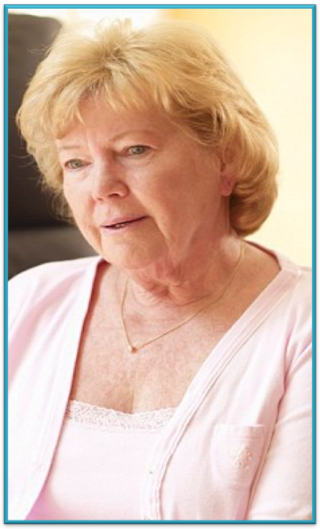

- Like to walk with her friends
- Enjoys doing house work such as vacuuming.
- Want to be able to keep on top of her COPD.
- Does not like getting sick as is looking for a way to be more pro-active when she does not feel as well.

Should Teresa have an action plan?

My COPD Action Plan Teresa (Patient's Name) Date May 25, 2015
Patient's Copy

Canadian Respiratory Guidelines

COPD Treatable. Preventable.

This is to tell me how I will take care of myself when I have a COPD flare-up.

My goals are To be able to do my everyday activities like walking with my friends and doing housework.

My support contacts are ______ (Name & Phone Number) and ______ (Name & Phone Number)

My Symptoms	I Feel Well	I Feel Worse	I Feel Much Worse URGENT
I have sputum.	My usual sputum colour is: Clear	Changes in my sputum, for **at least** 2 days. Yes ☒ No ☐ OR	My symptoms are not better after taking my flare-up medicine for 48 hours.
I feel short of breath.	When I do this: If I exercise too much like running to catch a bus.	More short of breath than usual for **at least** 2 days. Yes ☒ No ☐	I am very short of breath, nervous, confused and/or drowsy, and/or I have chest pain.

My Actions	Stay Well	Take Action	Call For Help
	I use my daily puffers as directed.	If I checked 'Yes' to one or both of the above, I use my **prescriptions** for COPD flare-ups.	I will call my support contact and/or see my doctor and/or go to the nearest emergency department.
	If I am on oxygen, I use ______L/min.	I use my daily puffers as usual. If I am **more** short of breath than usual, I will take 2 puffs of Ventolin up to a **maximum** of 4 times per day.	**I will dial 911.**
		I use my breathing and relaxation methods as taught to me. I pace myself to save energy.	**Important information:** I will tell my doctor, respiratory educator, or case manager **within 2 days** if I had to use any of my flare-up prescriptions. I will also make follow-up appointments to review my COPD Action Plan twice a year.
		If I am on oxygen, I will increase it from ___ L/min to ___ L/min.	

Notes:

THE LUNG ASSOCIATION™ L'ASSOCIATION PULMONAIRE — CANADIAN THORACIC SOCIETY / SOCIÉTÉ CANADIENNE DE THORACOLOGIE

Produced in collaboration with the *COPD & Asthma Network of Alberta (CANA).* *The Canadian Thoracic Society (CTS) acknowledges the past contributions of Living well with COPD and the Family Physician Airways Group of Canada.* PART 1 OF 2

My COPD Action Plan Teresa (Patient's Name) Date May 25, 2015
Physician's Copy

Canadian Respiratory Guidelines

COPD Treatable. Preventable.

This is to tell me how I will take care of myself when I have a COPD flare-up.

My goals are To be able to do my everyday activities like walking with my friends and doing housework.

My support contacts are ______ (Name & Phone Number) and ______ (Name & Phone Number)

My Symptoms	I Feel Well	I Feel Worse	I Feel Much Worse URGENT
I have sputum.	My usual sputum colour is: Clear	Changes in my sputum, for **at least** 2 days. Yes ☒ No ☐ OR	My symptoms are not better after taking my flare-up medicine for 48 hours.
I feel short of breath.	When I do this: If I exercise too much like running to catch a bus.	More short of breath than usual for **at least** 2 days. Yes ☒ No ☐	I am very short of breath, nervous, confused and/or drowsy, and/or I have chest pain.

My Actions	Stay Well	Take Action	Call For Help
	I use my daily puffers as directed.	If I checked 'Yes' to one or both of the above, I use my **prescriptions** for COPD flare-ups.	I will call my support contact and/or see my doctor and/or go to the nearest emergency department.
	If I am on oxygen, I use ______L/min.	I use my daily puffers as usual. If I am **more** short of breath than usual, I will take 2 puffs of Ventolin up to a **maximum** of 4 times per day.	**I will dial 911.**
		I use my breathing and relaxation methods as taught to me. I pace myself to save energy.	**Important information:** I will tell my doctor, respiratory educator, or case manager **within 2 days** if I had to use any of my flare-up prescriptions. I will also make follow-up appointments to review my COPD Action Plan twice a year.
		If I am on oxygen, I will increase it from ___ L/min to ___ L/min.	

Notes:

THE LUNG ASSOCIATION™ L'ASSOCIATION PULMONAIRE — CANADIAN THORACIC SOCIETY / SOCIÉTÉ CANADIENNE DE THORACOLOGIE

Produced in collaboration with the *COPD & Asthma Network of Alberta (CANA).* *The Canadian Thoracic Society (CTS) acknowledges the past contributions of Living well with COPD and the Family Physician Airways Group of Canada.* PART 1 OF 2

My COPD Action Plan Teresa ____________________ Date May 25, 2015
Educator's Copy (Patient's Name)

Canadian Respiratory Guidelines — COPD Treatable. Preventable.

This is to tell me how I will take care of myself when I have a COPD flare-up.

My goals are To be able to do my everyday activities like walking with my friends and doing housework.

My support contacts are ____________________ (Name & Phone Number) and ____________________ (Name & Phone Number)

My Symptoms	I Feel Well	I Feel Worse	I Feel Much Worse — URGENT
I have sputum.	My usual sputum colour is: Clear	Changes in my sputum, for **at least** 2 days. *Yes* ☑ *No* ☐ OR	My symptoms are not better after taking my flare-up medicine for 48 hours.
I feel short of breath.	When I do this: If I exercise too much like running to catch a bus.	More short of breath than usual for **at least** 2 days. *Yes* ☑ *No* ☐	I am very short of breath, nervous, confused and/or drowsy, and/or I have chest pain.
**My Actions**	**Stay Well**	**Take Action**	**Call For Help**
	I use my daily puffers as directed.	If I checked 'Yes' to one or both of the above, I use my **prescriptions** for COPD flare-ups.	I will call my support contact and/or see my doctor and/or go to the nearest emergency department.
	If I am on oxygen, I use ______L/min.	I use my daily puffers as usual. If I am **more** short of breath than usual, I will take 2 puffs of Ventolin up to a **maximum** of 4 times per day.	**I will dial 911.**
**Notes:**		I use my breathing and relaxation methods as taught to me. I pace myself to save energy. If I am on oxygen, I will increase it from ___ L/min to ___ L/min.	**Important information:** I will tell my doctor, respiratory educator, or case manager **within 2 days** if I had to use any of my flare-up prescriptions. I will also make follow-up appointments to review my COPD Action Plan twice a year.

THE LUNG ASSOCIATION™ L'ASSOCIATION PULMONAIRE — CANADIAN THORACIC SOCIETY / SOCIÉTÉ CANADIENNE DE THORACOLOGIE

Produced in collaboration with the *COPD & Asthma Network of Alberta (CANA).*
The Canadian Thoracic Society (CTS) acknowledges the past contributions of Living well with COPD and the Family Physician Airways Group of Canada.

My COPD Action Plan Teresa ____________________ Date May 25, 2015
Patient's Copy (Patient's Name)

Canadian Respiratory Guidelines — COPD Treatable. Preventable.

This is to tell me how I will take care of myself when I have a COPD flare-up.

My goals are to keep my lungs in good shape

My support contacts are my husband (764-1087) (Name & Phone Number) and ____________________ (Name & Phone Number)

Prescriptions for COPD flare-up (Patient to take to pharmacist as needed for symptoms)

These prescriptions may be refilled two times each, as needed, for 1 year, to treat COPD flare-ups. Pharmacists may fax the doctor's office once any part of this prescription has been filled.

Teresa Kelly — Patient's Name

123 456 789 101 213 — Patient Identifier (e.g. DOB, PHN)

1. (A) If **the colour** of your sputum **CHANGES**, start antibiotic Amoxicillin Dose: 500mg #pills: 1
   How often three times daily for #days: 10

   (B) If the first antibiotic was taken for a flare-up in the **last 3 months**, use this different antibiotic instead:
   Start antibiotic Moxifloxacin Dose: 400mg #pills: 1
   How often once daily for #days: 10

AND / OR

2. If you are **MORE short of breath** than usual, start prednisone ________ Dose: 50mg #pills: 10
   How often: once daily for #days: 5

Once I start any of these medicines, **I will tell** my doctor, respiratory educator, or case manager within **2 days**.

Dr. Dinkhout — Doctor's Name
892-3452 — Doctor's Fax
____________ — Doctor's Signature

123-567 — License
May 25, 2015 — Date

THE LUNG ASSOCIATION™ L'ASSOCIATION PULMONAIRE — CANADIAN THORACIC SOCIETY / SOCIÉTÉ CANADIENNE DE THORACOLOGIE

Produced in collaboration with the *COPD & Asthma Network of Alberta (CANA).*
The Canadian Thoracic Society (CTS) acknowledges the past contributions of Living well with COPD and the Family Physician Airways Group of Canada. PART 2 OF 2

Lignes directrices
canadiennes en santé respiratoire

COPD ACTION PLAN (Patient's copy)

Why do I need this COPD Action Plan?
- Your Action Plan is a written contract between you and your health care team. It will tell you how to manage your COPD flare-ups. Use it along with any other information you get from your health care team about managing your COPD every day.
- Your Action Plan will help you and your caregivers to quickly recognize and act to treat your flare-ups. This will keep your lungs and you as healthy as possible.

How will I know that I am having a COPD "flare-up"?
- You will often see a change in your amount or colour of sputum and/or you may find that you are more short of breath than usual. Other symptoms can include coughing and wheezing more.
- Your flare-up Action Plan is to be used only for COPD flare-ups. Remember that there are other reasons you may get short of breath, such as when you have pneumonia, are anxious, or have heart problems.
- Before or during a flare-up you may notice changes in your mood, such as feeling down or anxious. Some people have low energy or feel tired before and during a COPD flare-up.

What triggers a "COPD flare-up"?
- A COPD flare-up can sometimes happen after you get a cold or flu, or when you are stressed and run down.
- Being exposed to air pollution and changes in the weather can also cause COPD flare-ups. To learn about the daily air quality in your area, visit Environment Canada's Air Quality Health Index (AQHI) website at www.ec.gc.ca/cas-aqhi/ and click on 'Your Local AQHI Conditions'. Ask your health care team about ways to avoid all possible triggers.

When should I use this COPD Action Plan?
- Your COPD Action Plan is used only for COPD flare-ups.
- Remember that there are other reasons you may get short of breath, such as when you have pneumonia, are anxious, or have heart problems. If you become more short of breath but don't have symptoms of COPD flare-up, see a doctor as soon as possible.

REMEMBER:
- Learn about your COPD from a respiratory educator, credible websites, such as www.lung.ca, and education programs.
- Take your regular daily medicine as prescribed.
- Don't wait more than 48 hours after the start of a COPD flare-up to take your antibiotic and/or prednisone medicines. See your pharmacist quickly to get your prescriptions for COPD flare-up.
- When you start an antibiotic, make sure that you finish the entire treatment.
- Quitting smoking and making sure that your vaccinations are up-to-date (for flu every year and for pneumonia at least once) will help prevent flare-ups.
- Be as active as possible. Inactivity leads to weakness, which may cause more flare-ups or flare-ups that are worse than usual. Ask your doctor about pulmonary rehabilitation and strategies to help reduce your shortness of breath and improve your quality of life.
- Follow up with your doctor within 2 days after using any of your prescriptions for a COPD flare-up.

MY NOTES AND QUESTIONS:

My COPD Action Plan Teresa (Patient's Name) Date May 25, 2015
Physician's Copy

Canadian Respiratory Guidelines
COPD Treatable. Preventable.

This is to tell me how I will take care of myself when I have a COPD flare-up.

My goals are to keep my lungs in good shape

My support contacts are my husband (764-1087) (Name & Phone Number) and ______ (Name & Phone Number)

Prescriptions for COPD flare-up (Patient to fill as needed for symptoms)

These prescriptions may be refilled two times each, as needed, for 1 year, to treat COPD flare-ups. Pharmacists may fax the doctor's office once any part of this prescription has been filled.

Teresa Kelly — Patient's Name
123 456 789 101 213 — Patient Identifier (e.g. DOB, PHN)

1. (A) If **the colour** of your sputum **CHANGES**, start antibiotic Amoxicillin Dose: 500mg #pills: 1
   How often three times daily for #days: 10

   (B) If the first antibiotic was taken for a flare-up in the **last 3 months**, use this different antibiotic instead:
   Start antibiotic Moxifloxacin Dose: 400mg #pills: 1
   How often once daily for #days: 10

AND / OR

2. If you are **MORE short of breath** than usual, start prednisone Dose: 50mg #pills: 10
   How often: once daily for #days: 5

Once I start any of these medicines, **I will tell** my doctor, respiratory educator, or case manager within **2 days.**

Dr. Dinkhout — Doctor's Name
892-3452 — Doctor's Fax
______ — Doctor's Signature

123-567 — License
May 25, 2015 — Date

THE LUNG ASSOCIATION™ L'ASSOCIATION PULMONAIRE
CANADIAN THORACIC SOCIETY SOCIÉTÉ CANADIENNE DE THORACOLOGIE

Produced in collaboration with the *COPD & Asthma Network of Alberta (CANA)*. *The Canadian Thoracic Society (CTS)* acknowledges the past contributions of *Living well with COPD* and the *Family Physician Airways Group of Canada.* PART 2 OF 2

COPD ACTION PLAN (Physician's copy)

Pharmacological Treatment

1. Short-acting (beta$_2$-agonists and anticholinergic) bronchodilators to treat wheeze and dyspnea. Continue all of your long acting bronchodilators or inhaled steroids as prescribed.
2. Prednisone (oral) → 25-50 mg once daily for 10 days for patients with moderate to severe COPD.
3. Antibiotic choice is prescribed based upon the presence of risk factors as below.
4. Severe AECOPD complicated by acute respiratory failure is a medical emergency. Consider consultation with an emergency specialist or respirologist.

Antibiotic Treatment Recommendations for Acute COPD Exacerbations[1,2]

Group	Probable Pathogens	First Choice	Alternatives for Treatment Failure
I, Simple<br>Smokers<br>FEV1 > 50%<br>≤ 3 exacerbations per year	H. influenzae<br>M. catarrhalis<br>S. pneumoniae	Amoxicillin, 2nd or 3rd generation cephalosporin, doxycycline, extended spectrum macrolide, trimethoprimsulfamethoxazole (in alphabetical order).	Fluoroquinolone<br>β-lact/ β-lactamase inhibitor
II, Complicated, as per I, plus at least one of the following should be present: FEV1<50% predicted; ≥4 exacerbations/year; ischemic heart disease; use home oxygen or chronic oral steroids; antibiotic use in the past 3 months.	As in group I, plus: Klebsiella spp. and other Gram-negative bacteria Increased probability of β-lactam resistance.	Fluoroquinolone<br>β-lact/ β-lactamase inhibitor<br>(in order of preference).	May require parenteral therapy.<br>Consider referral to a specialist or hospital.
III, Chronic Suppurative<br>II, plus: Constant purulent sputum; some have bronchiectasis; FEV1 usually <35% predicted; chronic oral steroid use; multiple risk factors.	As in group II, plus: P. Aeruginosa and multi-resistant Enterobacteriaceae.	Ambulatory - tailor treatment to airway pathogen; P. Aeruginosa is common (ciprofloxacin)<br>Hospitalized - parenteral therapy usually required.	

General Recommendations for the Physician

- Patients need to be instructed to call or visit their treating physician if symptoms persist or worsen after 48 hrs in spite of patient-initiated treatment. Please instruct patients to notify their doctor, respiratory educator, or case manager within 2 days of filling any of their prescriptions for a COPD flare-up.
- Prescriptions for antibiotics and prednisone can be refilled twice each, as needed, for 1 year. Pharmacists may fax the doctor's office after any portion of the prescriptions for COPD flare-up has been filled.
- To reduce the risk of antibiotic resistance, if more than one treatment is required over 3 months, the class of antibiotics should be changed on subsequent courses of therapy.
- Review with your patient measures to prevent future COPD exacerbations including smoking cessation, annual influenza vaccination, pneumococcal vaccination and appropriate use of inhaled daily medications.
- Consider referral to a local respiratory educator and pulmonary rehabilitation program if available.

1 O'Donnell DE, Hernandez P, Kaplan A, Aaron S., et al. CTS recommendations for management of COPD – 2008 update – highlights for primary care. Can Resp J 2008; 15(Suppl A):1A-8A.

2 Balter MS, La Forge J, Low DE, Mandell L., et al. Canadian guidelines for the management of acute exacerbation of chronic bronchitis. Can Respir J 2003; 10(Suppl B):3B-32B.

Canadian Respiratory Guidelines

My COPD Action Plan Teresa (Patient's Name) Date May 25, 2015

Pharmacist's Copy

Canadian Respiratory Guidelines

COPD Treatable. Preventable.

This is to tell me how I will take care of myself when I have a COPD flare-up.

My goals are to keep my lungs in good shape

My support contacts are my husband (764-1087) (Name & Phone Number) and ____ (Name & Phone Number)

Prescriptions for COPD flare-up (Patient to fill as needed for symptoms)

These prescriptions may be refilled two times each, as needed, for 1 year, to treat COPD flare-ups. Pharmacists may fax the doctor's office once any part of this prescription has been filled.

Teresa Kelly — Patient's Name

123 456 789 101 213 — Patient Identifier (e.g. DOB, PHN)

1. (A) If **the colour** of your sputum **CHANGES**, start antibiotic Amoxicillin Dose: 500mg #pills: 1
   How often three times daily for #days: 10

   (B) If the first antibiotic was taken for a flare-up in the **last 3 months**, use this different antibiotic instead:
   Start antibiotic Moxifloxacin Dose: 400mg #pills: 1
   How often once daily for #days: 10

AND / OR

2. If you are **MORE short of breath** than usual, start prednisone ____ Dose: 50mg #pills: 10
   How often: once daily for #days: 5

Once I start any of these medicines, **I will tell** my doctor, respiratory educator, or case manager within **2 days**.

Dr. Dinkhout — Doctor's Name

892-3452 — Doctor's Fax

____ — Doctor's Signature

123-567 — License

May 25, 2015 — Date

THE LUNG ASSOCIATION™ L'ASSOCIATION PULMONAIRE

CANADIAN THORACIC SOCIETY / SOCIÉTÉ CANADIENNE DE THORACOLOGIE

Produced in collaboration with the COPD & Asthma Network of Alberta (CANA). The Canadian Thoracic Society (CTS) acknowledges the past contributions of Living well with COPD and the Family Physician Airways Group of Canada.

Framework for Pharmacist Role

1. Does the patient have undiagnosed COPD?
2. Is the patient properly managed by the current regimen?
3. **Is the patient properly using the device or using the right device?**
4. What pharmacist's services can aid COPD management?

Assessment of Inhalation Technique[1]

94% of patients committed at least one error

PRACTICE PEARL[2]
Review how to use various inhaler devices by reviewing creditable online demonstration videos or online instruction guides.

98% of patients claimed to know how to use their inhaler

1. Self TH, et al. Journal of Family Practice. 2011;60(12):714-720. 2. Canadian Lung Association. http://www.lung.ca/diseases-maladies/help-aide/devices-dispositifs/index_e.php.

Comparison of Inhaler Devices[1,2]

Devices	Advantages				
	Simple device setup [illegible]	Dose protected [illegible]	No spacer device [illegible]	Little lung-hand [illegible]	Can be used by patients with limited [illegible]s
Nebu[illegible]	[illegible]	[illegible]	[illegible]	[illegible]	[illegible]
Metered-dose inhaler (MDI)	✓	✓	✗	✗	✓
Pre-l[illegible] powd[illegible] inhal[illegible]	[illegible]	[illegible]	[illegible]	[illegible]	[illegible]
Dry p[illegible] inhaler (DPI) with dose caps[illegible]	✗	✗	✓	✓	✗
Soft [illegible] inha[illegible]	[illegible]	[illegible]	[illegible]	[illegible]	[illegible]

PRACTICE PEARL
Aid in tailoring the choice of inhaler device to the individual patient.

PRACTICE PEARL
Review inhaler technique at regular visits, allowing the patient to demonstrate and ask questions.

PRACTICE PEARL
Collaborate with the patient and physician to initiate an alternative inhaler device within the same therapeutic class if the patient is struggling to properly use the current inhaler device.

1. Newman, SP. Eur Respir Rev. 2005;14(96):102–108 2. Johns R. Pharm Prac. 2013 .

Metered Dose Inhalers

(Not an Exhaustive List)

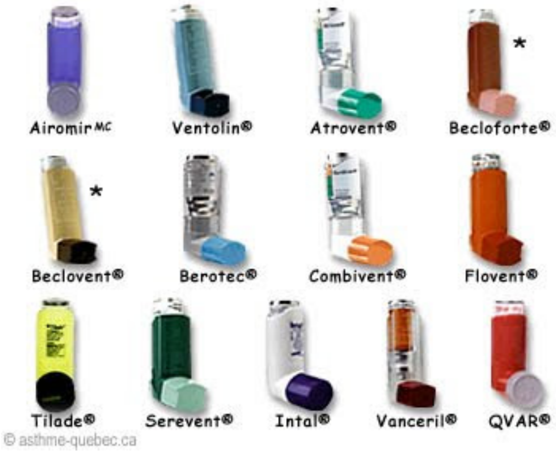

Metered Dose Inhaler
(MDI)

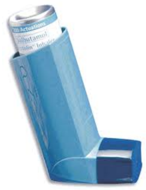

PRO'S	CON'S
Familiar	Should be used with spacer
Portable	Hard to co-ordinate breath
Inexpensive	No dose indicator
Quick relief	Need to shake to prime for each use

Spacers for use with MDIs

Spacers should be considered for use with MDIs

- Decreases deposition in mouth and throat
- Increases deposition in the lungs
- Reduces need for actuation/inhalation co-ordination
- May help some with dexterity problems
- Auditory warning whistle if inhalation too fast/strong

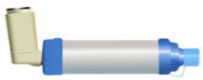

Multi Dose Inhaler (MDI)

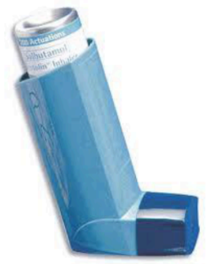

1. Remove cap from inhaler.
2. Shake the inhaler well 5-6 times.
3. Breathe out all the way.
4. Hold the inhaler upright, place the mouthpiece between your teeth and seal your lips around it.
5. As you start to inhale slowly, press the inhaler canister down to release a puff of medicine. Continue to breathe in slowly all the way.
6. Hold your breath for 5-10 seconds.
7. If you need another puff, wait 30 -60 seconds, then repeat all steps.
8. Put the cap back on when finished.
9. Rinse, gargle and spit after taking an inhaled steroid.

The Lung Association. www.on.lung.ca

MDI and Spacer

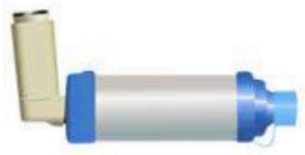

1. Remove the caps from the inhaler and spacer.
2. Shake the inhaler well 5-6 times before each puff.
3. Keep the inhaler upright and insert the mouthpiece into the back of the spacer.
4. Holding the spacer with one hand, place the spacer mouthpiece between the teeth and seal the lips around it.
5. Breathe all the way out. (You may prefer to breathe out before placing the mouthpiece into your mouth.)
6. As you start to inhale slowly, press the inhaler canister down with your other hand to release a puff of medicine into the spacer. Continue to breathe in **slowly** all the way.
7. Hold your breath for 5-10 seconds with the spacer in or out of your mouth.
8. (if you cannot take a slow deep breath, it is okay to breathe in as deeply as you can 2-3 times, keeping a good seal on the mouthpiece.
9. If another puff is needed, wait 30-60 seconds and repeat steps 2-7.

The Lung Association. www.on.lung.ca

Diskus
GSK

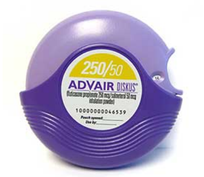

Trade Name	Flovent	Serevent	Advair
Generic Name	Fluticasone	Salmeterol	Fluticasone/ salmeterol
Class	ICS	LABA	ICS/LABA combo
Approval	2002	2002	2002

Diskus
GSK

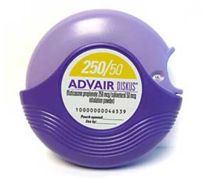

PRO'S	CON'S
Breath-actuated	Bulky
Propellant free	Sometimes only one step completed
Multi dose with counter	Moisture sensitive
Easy 2 step process	BID
No chamber necessary	Requires high inspiratory flow rate
No coordination required for actuation and inhalation	Strong and fast inhalation

Diskus

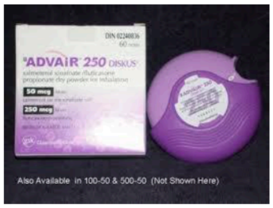

1. To open the inhaler, hold it in one hand.
2. Put the thumb of the other hand into tee thumb grip and slide it back until a click is heard. You will now see the mouthpiece and medicine lever.
3. With the mouthpiece facing you, slide the medicine lever back all the way until a click is heard.
4. (Do not shake it, tip it, drop it or breathe into it.)
5. Breathe out.
6. Seal your lips around the mouth piece and breathe in quickly and deeply, through your mouth.
7. Hold your breath for 5-10 seconds.
8. Close the inhaler by sliding the thumb grip back towards you as far as it goes, to its original position. You will no longer see the mouthpiece or medicine lever.
9. If you need another dose, repeat steps 1-7.

The Lung Association. www.on.lung.ca

HandiHaler
Boehringer Ingelheim

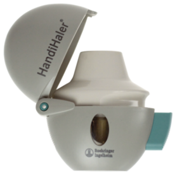

Trade Name	Spiriva
Generic Name	Tiotropium
Class	LAMA
Approval	Nov 2002

HandiHaler
Boehringer Ingelheim

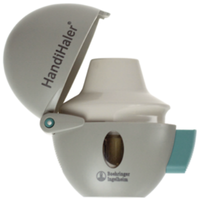

PRO'S	CON'S
Breath-actuated	Multiple steps
Portable	Requires good dexterity
Propellant free	
No activation/inhalation coordination required	
Easy to track remaining doses	

HandiHaler

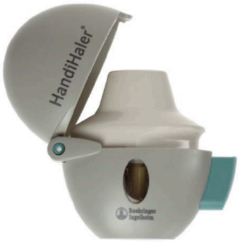

1. Open cap
2. Open mouthpiece
3. Remove capsule from blister and place in chamber
4. Close mouthpiece until it clicks
5. Press green piercing button in once and release
6. Breathe out gently away from mouthpiece
7. Put mouthpiece between teeth without biting and close lips to form good seal
8. Breathe in slowly and deeply, so capsule vibrates
9. Continue to breathe in as long as comfortable
10. While holding breath, remove inhaler from mouth
11. Breathe out gently away from mouthpiece
12. Put mouthpiece back between teeth without biting and close lips to form good seal
13. Breathe in slowly and deeply again, so capsule vibrates
14. Continue to breathe in as long as comfortable
15. While holding breath, remove inhaler from mouth
16. Breathe out gently away from mouthpiece
17. Open mouthpiece and remove used capsule
18. If an extra dose is needed, repeat steps 3 to 17
19. Close mouthpiece and cap

The Lung Association. www.on.lung.ca

Turbuhaler
Astra Zeneca

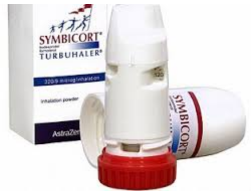

PRO'S	CON'S
Breath-actuated	Double turn confusing
Portable	Priming necessary
Multi-dose	Moisture sensitive
No chamber necessary	
No coordination required for inhalation	

Turbuhaler
Astra Zeneca

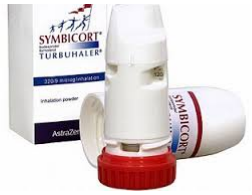

Trade Name	Pulmicort	Oxeze	Symbicort
Generic Name	Budesonide	Formoterol	Budesonide/ formoterol
Class	ICS	LABA	ICS/LABA combo
Approval	1996	1998 * Asthma	2002* asthma 2009 COPD

Turbuhaler

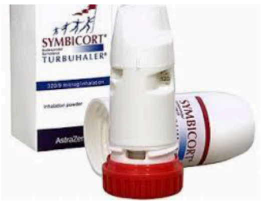

1. Hold it upright , with the grip at the bottom, unscrew and remove the cover.
2. Turn the grip all the way in one direction, then back all the way in the other direction. You will hear a click during this step which means the medicine is loaded.
3. (Do not shake, tip, drop or breathe into the turbuhaler.)
4. Breathe out away from the inhaler.
5. Place the mouthpiece between your teeth and seal your lips around it.
6. Breathe in quickly and deeply.
7. Hold your breath for 5 – 10 seconds.
8. If a second dose is needed, repeat steps 2-6.
9. When finished, replace the cover and twist until it is tightly closed.

The Lung Association. www.on.lung.ca

Breezhaler

Novartis

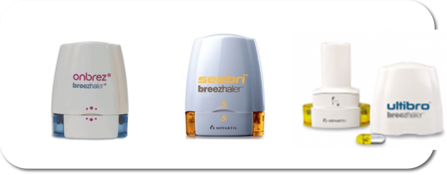

PRO'S	CON'S
Low resistance	Single dose inhaler
Small	Loose cap
Clear Capsules	Low resistance can lead to throat pooling
No coordination required for actuation and inhalation	Small
	Multiple piercing can lead to issues

Breezhaler

Novartis

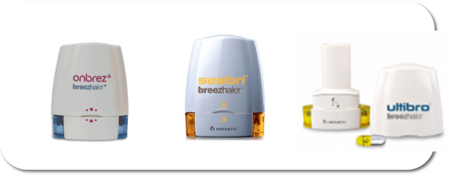

Trade Name	Onbrez	Seebri	Ultibro
Generic Name	indacaterol	glycopyrronium	inda/glyco combo
Class	LABA	LAMA	LABA/LAMA combo
Approval	Dec 2011	October 2012	Dec 2013

Breezhaler[1,2]

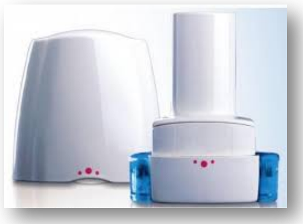

How to Use the Breezhaler Inhaler

1. Pull off the cap
2. Open the mouthpiece.
3. Remove capsule from package.
4. Place capsule in chamber.
5. Close mouthpiece until you hear it click.
6. Press side buttons once and release.
7. Breath out.
8. Seal lips around mouthpiece.
9. Breath in quickly and deeply.
10. Hold breath for 5-10 seconds.
11. Breath out.
12. Open the mouthpiece.
13. If capsule is not empty, repeat steps.
14. Open mouthpiece.
15. Remove empty capsule.
16. Replace cap.
17. Wash hands.

1. Young D etal. Indacaterol. p. 117-128.

Genuair
Astra Zeneca

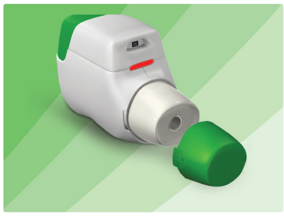

PRO'S	CON'S
Pre-loaded for one month	High resistance = hard for patients
Auditory cue	Loose cap
Large dose counter	Gritty texture in mouth
Easy to load dose	Bitter taste
No coordination required for actuation and inhalation	Powder deposition in mouth

Genuair
Astra Zeneca

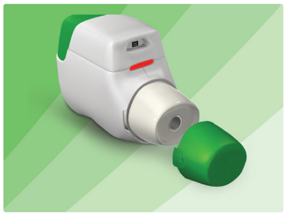

Trade Name	Tudorza
Generic Name	aclidinium
Class	LAMA
Approval	July 2013

Genuair[1,2]

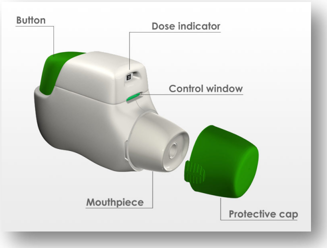

How to Use the Genuair Inhaler

1. Remove the protective cap by lightly squeezing the arrows and pulling outwards.
2. Hold horizontally with the green button facing up.
3. Press the green button all the way down.
4. Release the green button.
5. Check the control window has turned green.
6. Breath out fully.
7. Seal lips around mouthpiece.
8. Breath in strongly and quickly. (Click will be heard if inhaler is being used correctly.)
9. Continue to breath in strongly and quickly.
10. Hold breath for 5-10 seconds
11. Ensure control window has turned red.
12. Replace cap.
13. Rinse mouth.

1. Almirall Sofotec. http://www.almirallsofotec.com/en/genuair_fWhat.html.

Ellipta
GSK

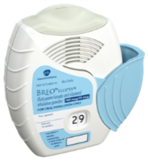

PRO'S	CON'S
Pre-loaded	High resistance
Very few steps	Bulky
Large dose counter	Must keep vents open
Attached cap	
No coordination required for actuation and inhalation	

Ellipta
GSK

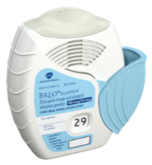

Trade Name	Breo	Anoro	Incruse
Generic Name	fluticasone/ vilanterol	umeclidinium/ vilanterol	umeclidinium
Class	ICS/LABA combo	LAMA/LABA combo	LAMA
Approval	July 2013	December 2013	April 2014

Ellipta[1,2,3]

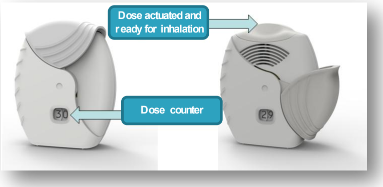

How to Use the Ellipta Inhaler

1. Hold the inhaler in an upright position.
2. Slide the cover down until you hear a click.
3. The dose counter will count down by one number. This shows that the dose is ready to inhale.
4. While holding the inhaler away from your mouth, breathe out as far as is comfortable.
5. Put the mouthpiece between your lips, and close your lips firmly around it.
6. Keep the tongue away from the mouthpiece.
7. Take one long, steady, deep breath in.
8. Hold this breath for at least 3-4 seconds.
9. Remove the inhaler from your mouth and breathe out slowly and gently.
10. Slide the cover upwards as far as it will go to recover the mouthpiece.
11. Rinse mouth if inhaler contains an ICS.

1. GlaxoSmithKline. http://hcp.gsk.ie/products/Anoro_Ellipta/the_ellipta_inhaler/how_to_use_ellipta.html. 2. Riley J et al. http://www.sec.gov/Archives/edgar/data/1080014/000110465913068858/a13-20084_1ex99d2.htm.

Respimat
Boehringer Ingelheim

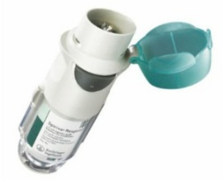

Trade Name	Spiriva	Combivent
Generic Name	tiotropium	ipratropium/ salbutamol
Class	LAMA	SAMA/SABA
Approval	Dec 2014	Jan 2014

Respimat
Boehringer Ingelheim

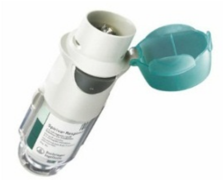

PRO'S	CON'S
Pre-loaded	Initial cartridge loading
Gentle inhalation experience	Priming necessary
Actuation independent of patient's inspiratory flow	
Minimal Inspiratory Flow rate required to deliver drug	
Attached cap	
Dose indicator	
High lung deposition / low oropharyngeal deposition	
Locking mechanism when cartridge is empty	

Respimat Soft Mist[1,2,3,4]

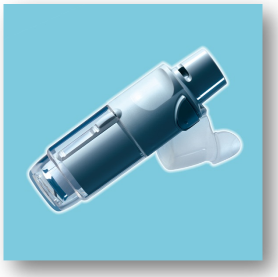

How to Use a Respimat Soft Mist Inhaler

1. Hold the inhaler upright with the cap closed.
2. Turn the transparent base until it clicks.
3. Open the cap.
4. Breathe out slowly.
5. Insert the mouthpiece.
6. Point the inhaler towards the back of the throat.
7. While taking a deep breath, press the dose-release button, and continue to breathe.
8. Hold your breath for 10 seconds, or as long as it is comfortable.
9. Then breathe out slowly.

1. Hodder R et al. International Journal of COPD 2009;4:225–232. 2. Boehringer Ingelheim. http://www.respimat.com/functions_and_use/how_to_use.html. 3. Boehringer Ingelheim. http://www.respimat.com/services/services/photographs.html.

Revisit Theresa

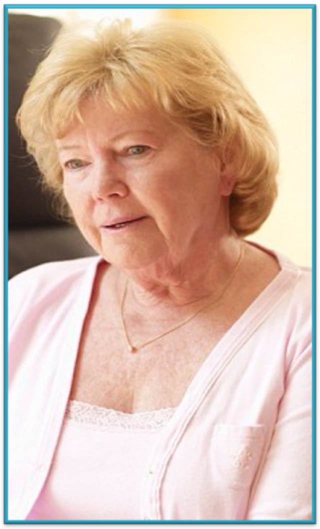

- Diagnosed with COPD
- Prescribed tiotropium once daily
- Shown how to use inhaler device
- Back for first refill
- Asked to demonstrate inhalation technique
- Not using inhaler device properly

What can the pharmacist do to improve inhalation technique?

What can the pharmacist do to improve Theresa's inhalation technique?

- ✔ Slowly re-demonstrate the correct technique
- ✔ Have Theresa repeat the demonstration, while checking against a checklist of essential steps
- ✔ Give Theresa a record of any step(s) incorrectly performed during her demonstration, by highlighting those on the checklist
- ✔ At subsequent visits, repeat assessment and education
- ✔ Direct Theresa to reliable information online that clearly shows her how to use her inhaler

1. National Asthma Council Australia. http://www.nationalasthma.org.au/uploads/content/237-Inhaler_technique_in_adults_with_asthma_or_COPD.pdf.

Framework for Pharmacist Role in COPD Management

1. Does the patient have undiagnosed COPD?
2. Is the patient properly managed by the current regimen?
3. Is the patient properly using the device or using the right device?
4. **What pharmacist's clinical services can aid COPD management?**

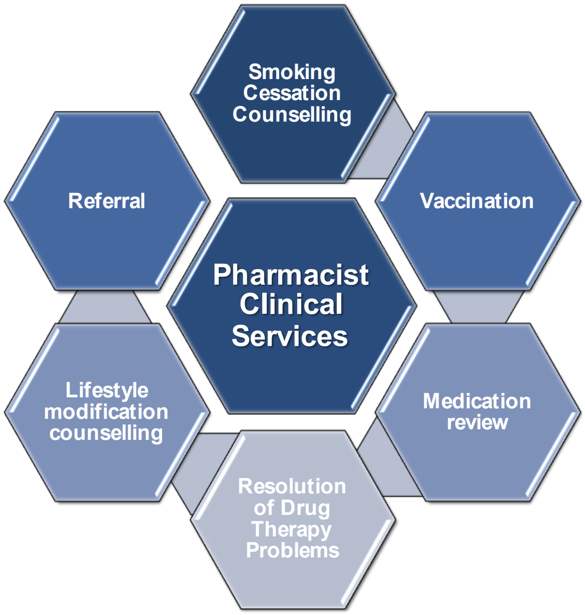

Smoking Cessation Counselling[1,2,3]

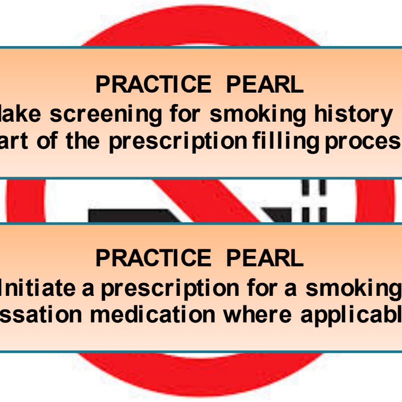

PRACTICE PEARL
Make screening for smoking history a part of the prescription filling process

PRACTICE PEARL
Initiate a prescription for a smoking cessation medication where applicable.

1. O'Donnell DE et al. Can Respir J. 2008; 15(Suppl A): 1A-8A. 2. O'Donnell DE et al. Can Respir J. 2007;14(Suppl B):5B-32B. 3. Fiore MC et al. Treating Tobacco Use and Dependence: 2008 Update. Clinical Practice Guideline. 2008.

Vaccination

Influenza[1]

- Role to play in prevention of acute exacerbations
- Reduces the morbidity and mortality from COPD by as much as 50% in elderly patients
- Reduces incidence of hospitalization by as much as 39% in patients with chronic lung disease

PRACTICE PEARL
When hosting vaccination shot clinics, contact all of your COPD patients to book an appointment.

Pneumococcal[1,2]

- [illegible] vaccine at least once
- High-risk patients should possibly receive vaccine again in five to ten years
- Pneumococcal 23-valent polysaccharide vaccine normally recommended for all adults ≥ 65 years

1. O'Donnell DE et al. Can Respir J. 2008; 15(Suppl A): 1A-8A. 2. Public Health Agency of Canada. http://www.phac-aspc.gc.ca/publicat/cig-gci/p04-pneu-eng.php.

Medication Review[1]

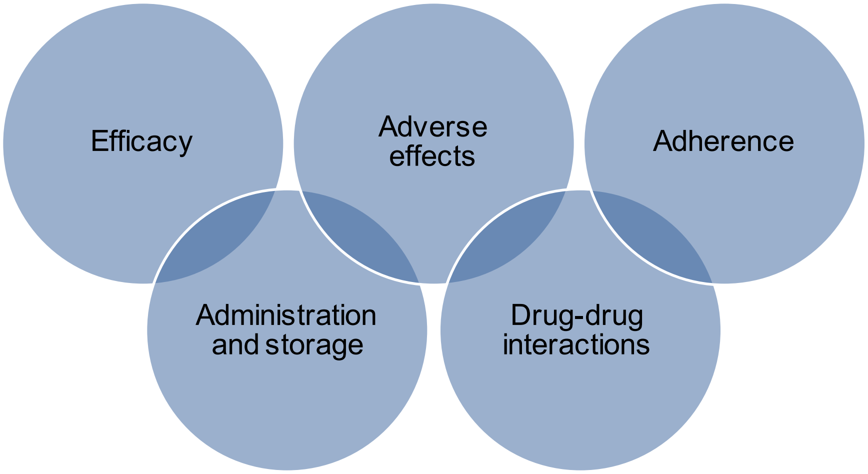

1. Bourbeau J et al. Thorax. 2008;63(9):831–838.

Resolution of Drug Therapy Problems

What?[1]

Identification by the

Possible impact?[1]

PRACTICE PEARL
When contacting the prescriber, make a clear clinical recommendation.

or when conducting a medication review

[illegible] therapy changed or adjusted (e.g. add therapy or discontinue therapy).

1. Pojakic N et al. OPA Conference; 2014.

Lifestyle Modification Counselling

Eating[1]

- Eat small frequent meals
- Breath evenly when chewing
- Take plenty of time to eat
- Reduce or avoid troublesome foods

Physical Activity[2,3]

- Regular activity to maintain
- Use slow, smooth movements
- Alternate periods of work and rest
- Pulmonary rehabilitation

PRACTICE PEARL
Engage patients in discussion of lifestyle modification options and encourage them to make decisions about which modifications will work best for them.

1. Canadian Lung Association. http://www.lung.ca/diseases-maladies/copd-mpoc/living-vivre/eat-manger_e.php. 2. Canadian Lung Association. http://www.lung.ca/diseases-maladies/copd-mpoc/living-vivre/energy-energie_e.php. 3. Canadian Lung Association. http://www.lung.ca/diseases-maladies/copd-mpoc/signs-signes/index_e.php.

Referral

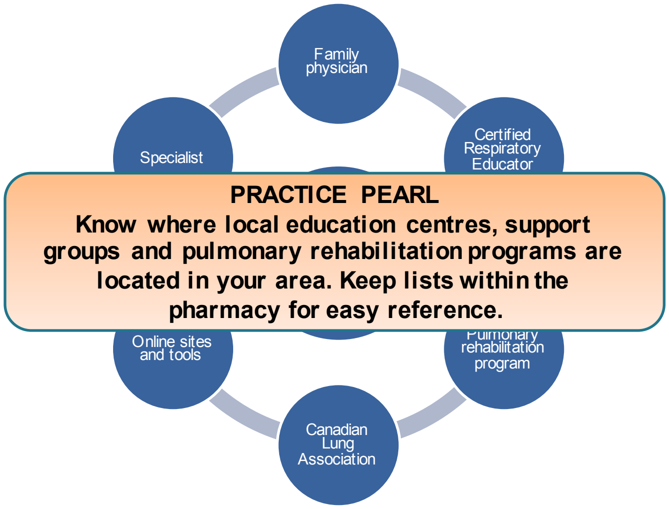

PRACTICE PEARL
Know where local education centres, support groups and pulmonary rehabilitation programs are located in your area. Keep lists within the pharmacy for easy reference.

1. Public Health Agency of Canada. http://www.phac-aspc.gc.ca/cd-mc/publications/copd-mpoc/ff-rr-2011-eng.php. 2. Canadian Lung Association. http://www.lung.ca/diseases-maladies/copd-mpoc/treatment-traitement/rehab-readaptation_e.php.

Key Takeaways

- Pharmacists are uniquely positioned to play an enhanced role in COPD medication and device management.
- Target at risk patients for spirometry to identify undiagnosed COPD cases, while increasing the likelihood of early and/or prompt diagnosis.
- Emphasize seriousness of the disease to all COPD patients and stress the importance of optimal symptom management and prevention of acute exacerbations.
- Promote the non-pharmacological and pharmacological management of COPD, focusing in particular on smoking cessation counselling, lifestyle modifications and influenza and pneumococcal immunization.

Key Takeaways

- Regularly assess whether COPD patients are optimally managed on current pharmacotherapy by assessing adherence, dyspnea and number and severity of AECOPDs.
- Discuss the advantages and disadvantages of various inhalation delivery devices/systems with patients and other healthcare providers.
- Assess inhalation technique at regular pharmacy visits as proper inhalation technique is just as important as the right drug in COPD management.
- Ensure patients have a COPD Action Plan and regularly review it with the patient.

Questions

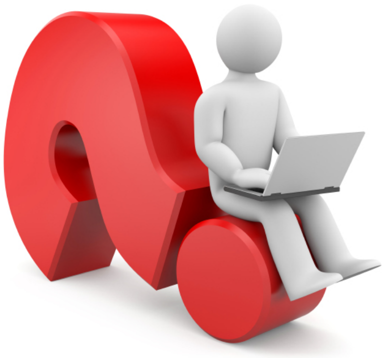

Supplement: Additional file 3: — COPD refresher presentation. The training presentation used to refresh participating pharmacists’ knowledge of COPD and its treatment. (PDF 6378 kb) [file 13063_2016_1623_MOESM3_ESM.pdf]
