# Supplementary material for: Effectiveness of a pharmacist-driven intervention in COPD (EPIC): study protocol for a randomized controlled trial
Source: Trials. 2016 Oct 13;17:502. doi: 10.1186/s13063-016-1623-7 (PMC5064938; doi:10.1186/s13063-016-1623-7)
Supplement: Additional file 4: — Intervention training presentation. The training presentation used to ensure all participating pharmacists are knowledgeable about and comfortable with the intervention used in the trial. (PDF 12947 kb) [file 13063_2016_1623_MOESM4_ESM.pdf]

Intervention Training

Effectiveness of a Pharmacist-Led Intervention in COPD (EPIC)

Intervention Overview

- The intervention will consist of the following:
  - Medication Review
    - Focusing on COPD medications
  - Patient Education
    - Inhaler technique
    - Non-pharm COPD options
  - COPD Action Plan
    - Developed with the patient and their primary HCP
  - Patient Referral
    - Smoking cessation
    - Pulmonary rehabilitation

Medication Review

Med Review

Detailed review of patient's medications:

- Efficacy
  - Focusing on COPD medications
  - As reviewed in COPD therapy presentation
- Safety
  - Focusing on COPD medications and interactions with other medications

Med Review – Medication Information

- Bronchodilators are medications that relax the airways, allowing you to breathe easier
- "Rescue inhalers" – these are short-term bronchodilators that start to work in ten minutes or less
- There are also long-acting treatments, usually taken once or twice a day, that bronchodilate over the course of the day

Med Review – Medication Information

- Inhaled corticosteroids are medications that reduce inflammation in the lungs, leading to better symptomatic control and reduced frequency of exacerbations
- Any corticosteroid inhalers – advise patient to wash out mouth after use

Med Review – Medication Information

- Combination products:
- There are different combo products available
- ICS/LABA – most common, shouldn't see ICS on its own for COPD as evidence is for combo
- Literature says reduced frequency of exacerbation
- LABA/LAMA – better symptomatic management
- May see others

Med Review – Medication Information

- Other medications:
- If patient is taking any other medications, make sure to go over those with the patient
- What the medication does (in a nutshell)
- What to expect – side effects, effect on disease

Med Review – Disease Information

Can review disease information as needed:

- Normally when we breath, air goes from our mouth or noses through the airways in our lungs into tiny air sacs, called alveoli
- These alveoli exchange oxygen in the air we breathe for carbon dioxide in our body, which we exhale

Med Review – Disease Information

- COPD usually results when an irritant, such as tobacco smoke, enters our lungs
- The irritant can cause inflammation in our lungs, resulting in damage to the airways and alveoli
- The airways can become thickened and scarred, limiting the way that air travels through them
- The alveoli can become damaged so that the normal oxygen exchange is impaired

Med Review – Disease Information

- The damage to the airways and alveoli results in common symptoms associated with COPD:
  - Coughing
  - Phlegm production
  - Shortness of breath, especially with activity
  - Fatigue

Med Review – Disease Information

- COPD is chronic, irreversible and progressive
- Medications help with symptoms, but the only thing that can affect lung function decline is quitting smoking
- COPD can lead to other diseases, like cardiovascular disease or osteoporosis

Med Review – Disease Information

- Exacerbations – **make sure patient understands what these are and how to recognize them**
  - A worsening of symptoms, which could include mucous production, shortness of breath, or cough, beyond what you would normally experience throughout the day

Med Review – Disease Information

- Exacerbations can be caused by infection, air pollutants, or sometimes just spontaneously
- Exacerbations can make your symptoms significantly worse
- They can accelerate lung function decline
- Medications can prevent exacerbations
- Early recognition and treatment is essential

Patient Education

Education – Inhaler Technique

- Go over device specifics with patient
- Encourage use of spacer devices
- Priming, taking a dose, storage, cleaning
- Get THEM to show YOU how to use the inhaler

- Inhalation device technique – manufacturer's website or Ontario Lung Association
- https://www.on.lung.ca/inhalationdevicevideos

Non-pharmacological Recommendations – Smoking Cessation

- Smoking is one of the most important interventions in the progression of COPD
- However, not everyone is ready to quit smoking
- Nicotine replacement in any form (patch, gum, etc) increases smoking abstinence rates

Non-pharmacological Recommendations – Smoking Cessation

- Other pharmacological therapy is also effective in helping patients quit smoking(champix, bupropion etc)
- Most effective – a combination of prescription medications and nicotine replacement therapies
- Evidence shows the more supportive counselling you put into it, the more patients get out of it[1]

Non-pharmacological Recommendations – Smoking Cessation[1]

- The 5 A's to help patients quit smoking:
- ASK: Systematically identify all tobacco users at every visit
- ADVISE: Strongly urge all tobacco users to quit
- ASSESS: Determine willingness to make a quit attempt
- ASSIST: Aid the patient in quitting
- ARRANGE: Schedule follow-up contact

Non-pharmacological Recommendations – Vaccinations[1]

- Influenza vaccine recommended for all patients
- Pneumococcal recommended for >65 years old or those with significant comorbidities
- Pharmacists in NL can administer both of these
- This makes it really easy to encourage their use to the patient as they can get it right at the pharmacy

Non-pharmacological Recommendations – Vaccinations

- Educate patient that getting vaccinated helps prevent viral and bacterial infections
- These infections lead to worsening symptoms and potential for exacerbation

Non-pharmacological Recommendations – Exercise[1]

- Physical exercise is safe and encouraged for people with COPD
- Exercise tolerance can be improved by medications
- Make sure that patients are aware of this – can associate in patient's mind improvement in quality of life with taking their medications properly

Non-pharmacological Recommendations – Exercise[1]

- Exercise is an important component in pulmonary rehab
- It can help improve dyspnea and fatigue symptoms in patients with COPD
- Recommend individuals walk to a symptom-limited maximum, rest, and then continue walking until 20 minutes of exercise

COPD Action Plan

COPD Action Plan

- Develop and review the CTS Action Plan with the patient
- This plan is focused on identifying exacerbations and making a plan for the patient in the event that an exacerbation does occur

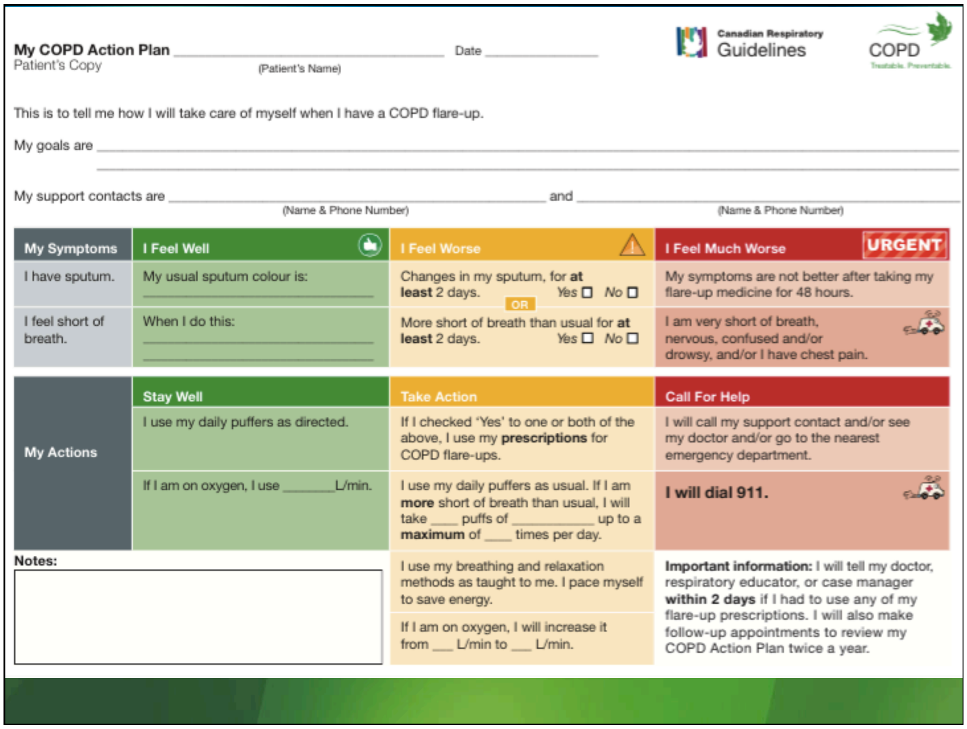

My COPD Action Plan ______________________ Date __________
Patient's Copy (Patient's Name)

Canadian Respiratory Guidelines
COPD Treatable. Preventable.

This is to tell me how I will take care of myself when I have a COPD flare-up.

My goals are ______________________

My support contacts are ______________________ (Name & Phone Number) and ______________________ (Name & Phone Number)

My Symptoms	I Feel Well	I Feel Worse	I Feel Much Worse URGENT
I have sputum.	My usual sputum colour is: ______	Changes in my sputum, for **at least** 2 days. Yes ☐ No ☐	My symptoms are not better after taking my flare-up medicine for 48 hours.
		OR	
I feel short of breath.	When I do this: ______ ______	More short of breath than usual for **at least** 2 days. Yes ☐ No ☐	I am very short of breath, nervous, confused and/or drowsy, and/or I have chest pain.

My Actions	Stay Well	Take Action	Call For Help
	I use my daily puffers as directed.	If I checked 'Yes' to one or both of the above, I use my **prescriptions** for COPD flare-ups.	I will call my support contact and/or see my doctor and/or go to the nearest emergency department.
	If I am on oxygen, I use ______L/min.	I use my daily puffers as usual. If I am **more** short of breath than usual, I will take ____ puffs of __________ up to a **maximum** of ____ times per day.	**I will dial 911.**

Notes:

I use my breathing and relaxation methods as taught to me. I pace myself to save energy.

If I am on oxygen, I will increase it from ___ L/min to ___ L/min.

Important information: I will tell my doctor, respiratory educator, or case manager **within 2 days** if I had to use any of my flare-up prescriptions. I will also make follow-up appointments to review my COPD Action Plan twice a year.

Patient Referral

Refer

- All
- Sor

e 4

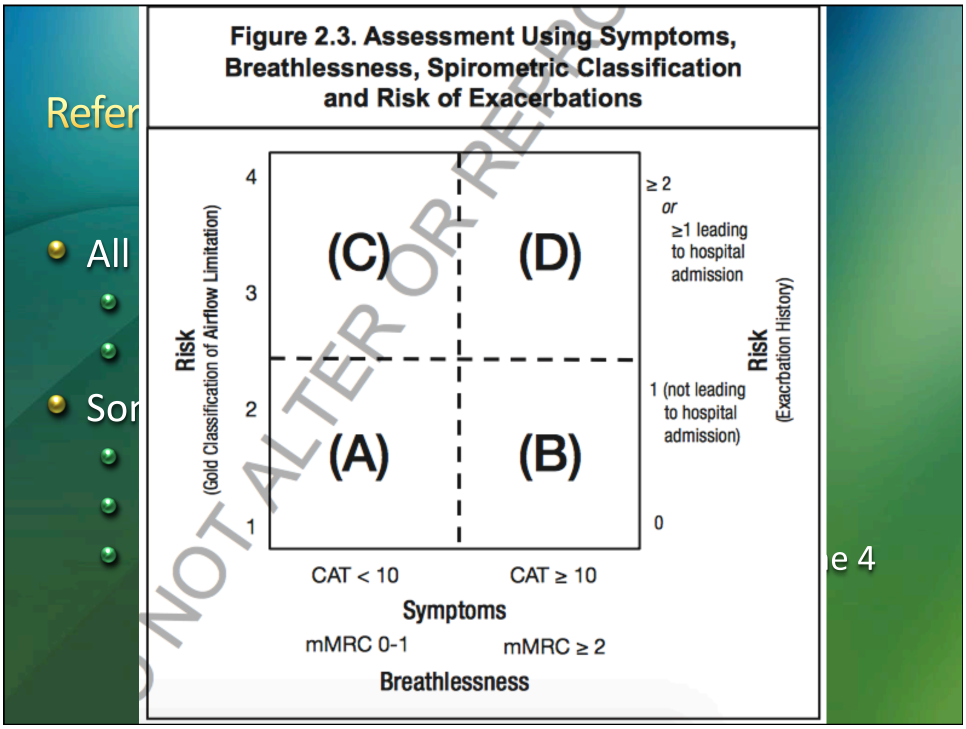

References

1. Global Initiative for Chronic Obstructive Pulmonary Disease. "Global Strategy for the Diagnosis, Management, and Prevention of Chronic Obstructive Pulmonary Disease." 2015.
2. UpToDate. (2014, January) Patient Information: Chronic Obstructive Pulmonary Disease (COPD), including emphysema (Beyond the Basics). Netherlands: Wolters Kluwer. Retrieved 17 July 2015. http://www.uptodate.com/contents/chronic-obstructive-pulmonary-disease-copd-including-emphysema-beyond-the-basics
3. UpToDate. (2014, June) Patient Information: Chronic Obstructive Pulmonary Disease (COPD), treatments (Beyond the Basics). Netherlands: Wolters Kluwer. Retrieved 17 July 2015. http://www.uptodate.com/contents/chronic-obstructive-pulmonary-disease-copd-treatments-beyond-the-basics
