# Supplementary material for: Effectiveness of a pharmacist-driven intervention in COPD (EPIC): study protocol for a randomized controlled trial
Source: Trials. 2016 Oct 13;17:502. doi: 10.1186/s13063-016-1623-7 (PMC5064938; doi:10.1186/s13063-016-1623-7)
Supplement: Additional file 5: — Study design presentation. The training presentation used to familiarize the participating pharmacists with the design and objectives of the trial. (PDF 18981 kb) [file 13063_2016_1623_MOESM5_ESM.pdf]

STUDY DESIGN

Increasing Quality of Life and Reducing Pulmonary Exacerbations in Chronic Obstructive Pulmonary Disease (COPD) by Providing Pharmacist-Driven Improvement in Medication Adherence: A Pragmatic Cluster Randomized Controlled Trial

Disclosures

- No relevant disclosures

Introduction

- Outline of the study
- Detailed Patient Flow
- Consent form and process
- Data collection forms

Study Background

- Pharmacist-led interventions have been shown to reduce hospitalizations and exacerbations
- Non-adherence rates are high in COPD, especially related to improper device technique

Study Background

- The purpose of this study is to evaluate a multifactorial pharmacist intervention to increase patient medication adherence, resulting in improved quality of life, fewer hospitalizations or exacerbations, and more effective use of health care expenditures

Study Design Overview

- A pragmatic cluster RCT
  - Pragmatic – real-life routine practice conditions[1]
  - Cluster RCT – Groups of individuals are randomized as opposed to the individuals themselves
    - In this study, the pharmacies will be randomized

Study Design Overview

- Each pharmacy will be randomized to either intervention or control group by the research team as they sign on
  - Each pharmacy will aim to recruit about 7 patients
- Control group pharmacies deliver "usual care" (more on this in a minute)
- Intervention group pharmacies will deliver the enhanced care intervention

Study Question

- **Population**: Patients >40yo with a diagnosis of COPD
- **Intervention**: Multifactorial pharmacist-led intervention
- **Comparison**: Usual care
- **Outcomes**:
  - Adherence
  - Quality of life
  - Inhaler technique
  - Healthcare resource utilization
  - Medication use

Inclusion Criteria

- Confirmed COPD by physician diagnosis
- Age >40
- Sufficient ability to answer questionnaires in English

Exclusion Criteria

- Known $FEV_1/FVC < 30\%$
- Diagnosis of dementia
- Prescription for cholinesterase inhibitors
- Known presence of terminal illness
- Patients who do not provide consent

Usual Care vs. Enhanced Care

- **Usual care** is considered the normal, safe and effective distribution of medications to patients
  - The pharmacist should respond to the patient, and whatever clinical information they have about them, as they otherwise would in practice
  - Because of this, the level of care may be up to intervention-level care
- **Enhanced care** will consist of medication reviews, targeted teaching, action plans, improving inhaler technique, adherence support strategies, and referral to other healthcare areas

Primary Outcome

- Primary outcome: Medication adherence
  - Medication Possession Ratio (MPR)
    - Ratio of days of medication supplied over the 6-month follow-up period
  - Moriskey Medication Adherence Scale (MMAS-8)

Secondary Outcomes

- Quality of life measured
  - St. George's Respiratory Questionnaire
- Medication inhalation technique
  - Device-specific checklist
- Healthcare resource utilization
  - frequency of physician visits/hospitalizations, use of pulmonary rehab
  - patient self-reporting using data collection forms

Secondary Outcomes

- Medication use
  - Antibiotic and corticosteroid use as an indicator of exacerbation history– patient self-reporting
- All outcomes will be scored through questionnaires found in the *Data Collection Form*

Detailed Study Patient Flow

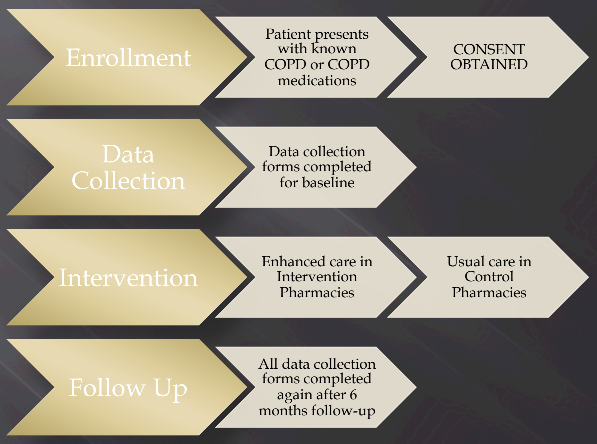

Step-by-Step

- Patient presents to pharmacy with COPD medications or known to have COPD
- Consult *Pharmacist Instructions* for a checklist of forms and procedures
- Ask patient if they are interested in participating and introduce patient to *Consent to Take Part in Research* form

Step-by-Step

- If yes, walk the patient through the consent process
- If no, patient care will resume as normal
- Fill out the *Patient Screening and Contact Information* form by contacting the patient and/or the patient's physician

Detailed Study Patient Flow

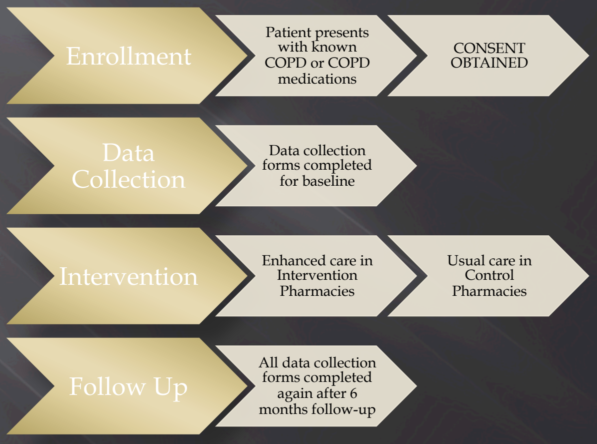

Step-by-Step

- If patient meets inclusion criteria, administer baseline questionnaires

Detailed Study Patient Flow

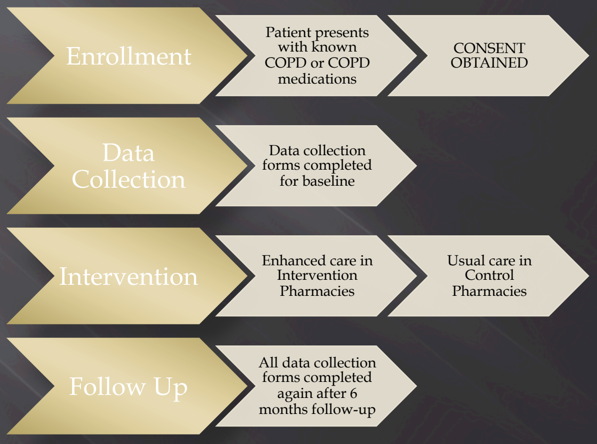

Step-by-Step

- Control patients given usual care
- Intervention patients given systematic pharmacist intervention (details soon!)

Detailed Study Patient Flow

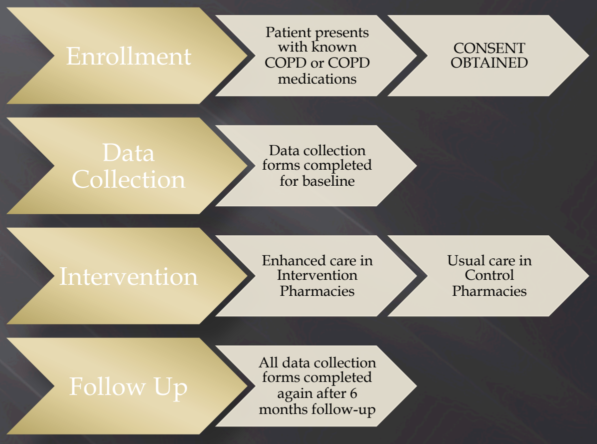

Forms and Other Materials

Pharmacist Instructions

- This is a checklist of all necessary steps and forms to guide patient flow
- Please complete tasks in order
- Please ensure that there are copies of each form listed on this checklist for each patient

Increasing Quality of Life and Reducing Pulmonary Exacerbations in Chronic Obstructive Pulmonary Disease (COPD) by providing pharmacist-driven improvement in medication adherence: A Pragmatic Cluster Randomized Controlled Trial

PHARMACIST INSTRUCTIONS

Participant Name:________________________

Each participant will require:

- Patient Consent Form
- Study Log
- Patient Screening and Contact Information Form
- Data Collection Form

Consent Form

- Different form for Control and Intervention
- All patients must give consent before participating in the study
- Patients must give consent before any information is acquired from patient OR physician
- The consent form outlines the study purpose as well as what the patient might expect from being involved in the study

Consent Form

- The consent form explains confidentiality, privacy, and potential risks and benefits for the patient
- The patient must read the consent form
- Ensure the patient understands the form and what it represents to give consent
- Answer any questions the patient may have
  - Direct them to me (Erin Davis 777-7232, emdavis@mun.ca) if necessary

MEMORIAL UNIVERSITY
School of Pharmacy
300 Prince Philip Drive, Health Sciences Centre
St. John's, NL, Canada A1B 3V6
Tel: 709-777- 8300 Fax: 709 777-8301
www.mun.ca/pharmacy

Consent to Take Part in Research

TITLE:
Increasing Quality of Life and Reducing Pulmonary Exacerbations in Chronic Obstructive Pulmonary Disease (COPD) by Providing Pharmacist-driven Improvement in Medication Adherence: A Pragmatic Cluster Randomized Control Trial

Patient Screening/Contact Information Form

- This form is to ensure that the patient is eligible for the study
- There are two sections:
  - 1. Eligibility Criteria
  - 2. Patient Contact Information

Patient Screening/Contact Information Form

- Gather any necessary inclusion/exclusion data from the patient or their physician, then fill out the eligibility checklist
- Once eligibility is confirmed, have patient fill out the Contact Information section

Increasing Quality of Life and Reducing Pulmonary Exacerbations in Chronic Obstructive Pulmonary Disease (COPD) by providing pharmacist-driven improvement in medication adherence: A Pragmatic Cluster Randomized Controlled Trial

Study# ___ ___ ___ - ___ ___ ___ - ___ ___ Randomization Code: ___ ___

Site number Subject number

PATIENT SCREENING AND CONTACT INFORMATION

SECTION 1: PATIENT SCREENING

For completion by the pharmacist

Increasing Quality of Life and Reducing Pulmonary Exacerbations in Chronic Obstructive Pulmonary Disease (COPD) by providing pharmacist-driven improvement in medication adherence: A Pragmatic Cluster Randomized Controlled Trial

SECTION II: CONTACT INFORMATION

For completion by study participant

Data Collection Form

- There are three main sections to this form:
  - Section I contains a list of the patient's COPD medications, as well as a summary of the patient's data
  - Section II is for the pharmacist and includes the method of scoring the MMAS and SGRQ and the device technique checklist

Data Collection Form

- Section III is for the patient to fill out and includes the actual questionnaires for the patient: the MMAS and SGRQ
  - Give this final section to the patient to fill out
- Using Section II, score the patient's results and put the final score in the summary in Section I
- Add the device technique checklist score to Section I

Data Collection Form

- There is a tick box at the top of the form indicating whether the form is being used at the start of the study or at the end of six months
- There will be **two copies** of the same form for each patient, one at baseline and one at 6 months
- At 6 months, there is an additional portion of Section I to fill out with health care utilization data

Increasing Quality of Life and Reducing Pulmonary Exacerbations in Chronic Obstructive Pulmonary Disease (COPD) by providing pharmacist-driven improvement in medication adherence: A Pragmatic Cluster Randomized Controlled Trial

Study# ___ ___ ___ - ___ ___ ___ - ___ ___ Randomization Code: ___ ___

Site number Subject number

SECTION I: DATA SUMMARY

To be completed by the Pharmacist

Increasing Quality of Life and Reducing Pulmonary Exacerbations in Chronic Obstructive Pulmonary Disease (COPD) by providing pharmacist-driven improvement in medication adherence: A Pragmatic Cluster Randomized Controlled Trial

SECTION II: QUESTIONNAIRE SCORING

Coding Instructions for the Morisky Medication Adherence Scale (8-item)

After the patient completes the Morisky Medication Adherence Scale, please score their answers using the following algorithm. When you are finished, record the score in SECTION I.

Increasing Quality of Life and Reducing Pulmonary Exacerbations in Chronic Obstructive Pulmonary Disease (COPD) by providing pharmacist-driven improvement in medication adherence: A Pragmatic Cluster Randomized Controlled Trial

SECTION III: PATIENT QUESTIONNAIRES

To be completed by the Patient

References

- 1. Patsopoulos NA. "A Pragmatic View on Pragmatic Trials."
- 2. McKenzie J, Ryan R, Di Tanna GL. Cochrane Consumers and Communication Review Group. "Cochrane Consumers and Communication Review Group: cluster randomised controlled trials" http://cccrg.cochrane.org, March 2014 (accessed 15 July 2015)
