# Supplementary material for: Effectiveness of a pharmacist-driven intervention in COPD (EPIC): study protocol for a randomized controlled trial
Source: Trials. 2016 Oct 13;17:502. doi: 10.1186/s13063-016-1623-7 (PMC5064938; doi:10.1186/s13063-016-1623-7)
Supplement: Additional file 6: — COPD pamphlet. An educational pamphlet on COPD management from the Canadian Lung Association. (PDF 1041 kb) [file 13063_2016_1623_MOESM6_ESM.pdf]

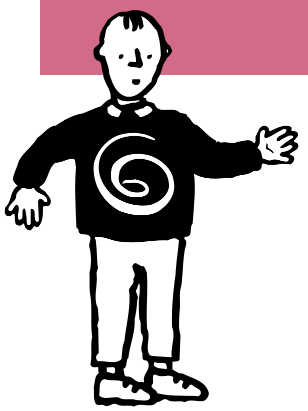

# ASK THE BREATHWORKS COACH

## I know I will always have COPD. What can I do to manage it?

COPD is a chronic disease, which means it cannot be cured. However, there are many ways you can manage COPD so that you can do the things you want to do.
To stay healthy, you need to understand your disease and learn how to manage it.

### Here is what you can do:

- quit smoking (most important)
- exercise regularly
- eat well
- get an annual flu shot each fall
- get a pneumonia shot
- wash your hands regularly
- take your medications properly
- learn how to live with COPD
- follow your action plan

### 1. Quit smoking

It is never too late to quit. Although quitting smoking will not fix the damage that has already been done to your lungs, it can help prevent more damage. Quitting smoking is hard but it is the best thing you can do to feel better.

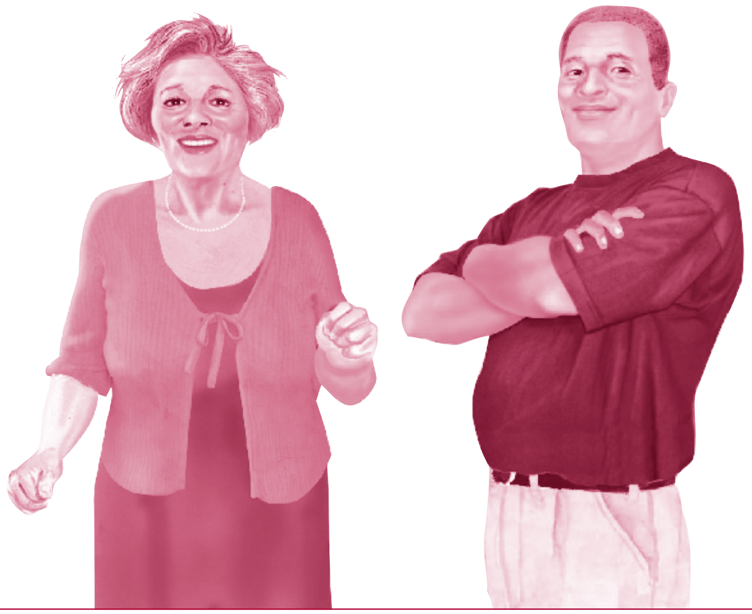

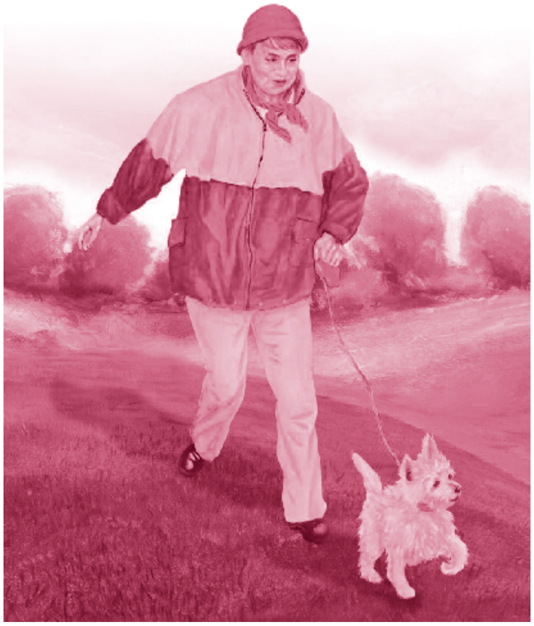

## 2. Exercise regularly

Regular exercise is important. If your muscles are in shape, they can work with less oxygen. That means you don't have to breathe in as much air to do the same amount of work. You are stronger and can do more before you feel tired. There are special exercise programs for people with COPD. These programs, called pulmonary rehabilitation programs, are run by health professionals who can help you find the exercise that you can do and enjoy. (To learn more about programs, check out The Lung Association fact sheet: **Pulmonary Rehabilitation**.) Contact your provincial Lung Association to find out if there is a pulmonary rehabilitation program in your area.

## 3. Eat well

By eating well, you will have more energy to breathe properly and do the things you want to do. Eating healthy foods will help you maintain your weight, feel good about yourself, and reduce your risk of serious health problems like diabetes and stroke.

### Tips for eating well:

- Eat a variety of foods, especially fruit, vegetables and whole grains.
- Avoid greasy food or junk foods.
- Limit salt, alcohol and caffeine.

For more information about eating well, refer to Canada's Food Guide. Developed by Health Canada, the Food Guide is available for download at http://www.hc-sc.gc.ca/fn-an/food-guide-aliment/order commander/index_e.html or you can order a copy by calling 1-800-O-Canada. If you have more specific questions about your diet, talk to a dietician.

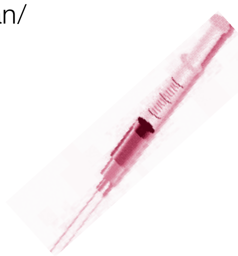

## 4. Get the flu shot

To help you to stay healthy, ask your doctor or health-care provider about getting a flu shot. This should be done every year in the fall. A person who has an allergy to eggs should not get the flu shot.

## 5. Get the pneumonia shot

Another way you can stay healthy is to get the pneumonia shot. The pneumonia shot is not given every year, but some people may need to repeat the pneumonia shot every five to ten years. Talk to your doctor or health-care provider about getting the pneumonia shot.

## 6. Wash your hands

Proper hand washing can help reduce your chances of getting a cold or the flu. Always wash your hands before eating or preparing a meal and after using the toilet.

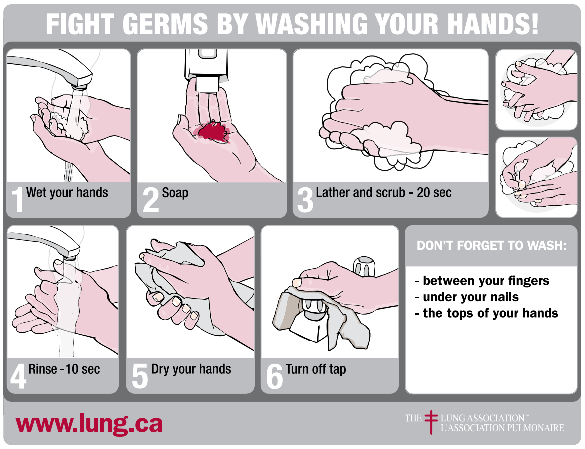


**Remember, stay healthy and follow your action plan.**

## 7. Take your medications properly

If your COPD medication is not getting in to your lungs, it cannot do its job. That is why it is important to follow your doctor's instructions exactly when taking any medications. Ask your respiratory educator or health-care provider to watch you use your medication device to make sure that your medication is getting in to your lungs.

## 8. Learn how to live with COPD

COPD affects almost everything you do. It may be hard to do some of the things that you used to enjoy. You may also get tired quickly. When you learn how to manage your COPD, you can enjoy the things you want to do. You can learn to pace yourself. Talk to your doctor or respiratory educator about ways to manage your COPD.

## 9. Follow your action plan

An action plan is a written set of instructions from your doctor. It explains what medication you should be taking on a daily basis when you feel well and how to increase your medication if your breathing problems get worse. Your action plan can help you to deal with any problems before they get worse.

# What can I do to manage flare-ups?

### What is a flare-up?

A flare-up is what happens when your COPD starts getting worse. You may have one or more of the following for 48 hours or longer:

- more shortness of breath than usual
- more coughing
- more mucus than usual
- mucus changes colour

### What causes a flare-up?

Flare-ups can be caused by:

- infections
- smoke
- dust
- allergens
- air pollution
- strong fumes or odours
- weather changes (cold air, hot air or humid air)
- stress

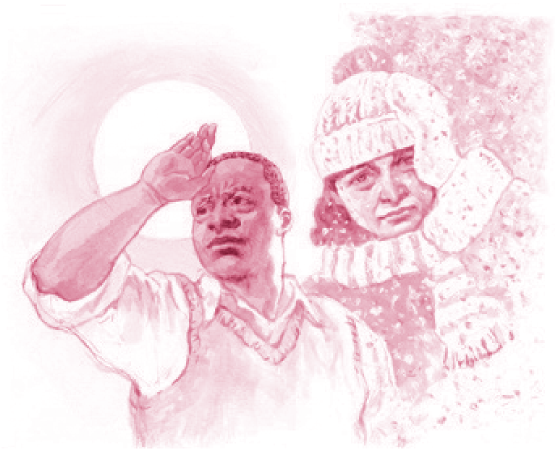

### How can I avoid flare-ups?

You can avoid flare-ups by learning what makes your COPD worse and what to do before it gets worse. For example, if cold air bothers you, cover your mouth and nose with a scarf (wrapped loosely). Another example may be to take your medication before walking on a windy day. Talk to your doctor about preparing an action plan. An action plan can help you recognize early signs of a flare-up and what steps you can take.

### What should I do if I start to have a flare-up?

Follow your action plan. It will tell you what to do if your breathing is getting worse, what medications to take and when to seek medical help.

Sometimes flare-ups still happen, despite your best efforts to prevent them. If you learn to recognize when the flare-up is starting, you may have time to start treatment before your flare-up hits full force. Early treatment could save you from having to stay in hospital.

**Remember, begin to manage your flare-up as early as possible.**

**Signs that the flare-up may be getting worse:**

- an increase in the thickness or stickiness of your mucus
- chest pain
- fever
- swollen ankles
- needing to sleep sitting up instead of lying down
- morning headaches, dizziness, trouble sleeping, confusion
- blue lips or fingers
- feeling sick

If you notice any of the above signs, call your doctor right away. If you can't reach your doctor, have someone drive you to the nearest emergency room.

If you are ever unsure about what to do, call your doctor and ask for guidance

**If you have any of the following, go to the emergency room immediately. Call 9-1-1 or an ambulance. Do not drive yourself.**

- sudden, extreme breathlessness
- sudden chest pain
- feeling confused, agitated or drowsy

## What can I expect at the hospital?

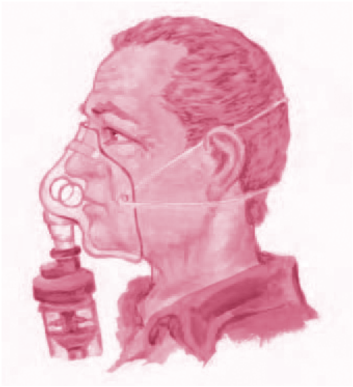

- You will be asked what medications you currently take so it is a good idea to always have an up-to-date list of all your medications (including how much you take and how often) that you can bring with you.
- You will be asked questions about your COPD.
- You will be given medications to open your airways so you can breathe easier.
- Your pulse, temperature and blood pressure will be taken.
- You may be given oxygen with a mask.
- An attachment may be placed on one of your fingers. This measures the oxygen in your blood.
- An intravenous or IV may be started. This provides another way of giving you medication to open your airways.
- You may be given an anti-inflammatory to decrease swelling in your airways.
- You may be given an antibiotic if your flare-up is due to a lung infection.

**What should I do before I leave the hospital?**

Make sure you understand any medication changes that have been made at the hospital. This includes medications that have been started or increased during your hospital stay. You should know how long to keep taking each medication and when you should decrease or stop taking them.

If the hospital staff thinks it will take more than a few days for your emergency room record to reach your doctor, it might be a good idea to ask for a copy to take home with you.

**What happens when I go home?**

Within 2-3 days of leaving the hospital, you should call your doctor for an appointment. You and your doctor need to talk about why you ended up in the emergency room so you can prevent it from happening again.

Your doctor will also need to know about any medications you were given, any new drugs or any increase in the dose of your usual medications. Your doctor can also tell you how long to keep taking the medication prescribed at the hospital before returning to your regular medication routine.

Your doctor may also want you to see a respiratory educator who can help you manage your COPD

**Remember, always keep an up-to-date list of your medications including how much you take and how often you take them.**

## COPD ACTION PLAN FOR ____________________

| CONTACT INFORMATION | |
|---|---|
| Name: ____________________<br>Dr.: ____________________<br>Hospital Emergency #: ____________________<br>Pharmacist (name): ____________________<br>Phone #: ____________________ | Phone Number: ____________________<br>BreathWorks Helpline: 1-866-717-2673<br>Respiratory Educator: ____________________<br>Phone #: ____________________ |

| I FEEL WELL | WHAT SHOULD I DO? |
|---|---|
| • My breathing problems have not changed (shortness of breath, cough, and mucus).<br>• My appetite is normal.<br>• I have no trouble sleeping.<br>• I am able to exercise and do my daily activities as usual. | Continue taking my medications as prescribed by my doctor.<br>____________________<br>____________________<br>____________________<br>____________________ |

| I FEEL DIFFERENT | WHAT SHOULD I DO? |
|---|---|
| • I am feeling stressed or have been exposed to things that make my breathing worse.<br>• I am more short of breath than usual.<br>• I am coughing or wheezing more than usual.<br>• I have more mucus than usual. | • Try to avoid or stay away from what is making my breathing worse.<br>• Breathe from my diaphragm or with pursed-lips.<br>• Lean forward. Relax my neck, shoulders and arms.<br>• If standing, lean against a wall with my feet slightly apart.<br>• Take my medications, especially my reliever.<br>____________________ |

| I FEEL DIFFERENT (Infection) | WHAT SHOULD I DO? |
|---|---|
| • I have increased shortness of breath.<br>• I have more mucus than usual.<br>• I have green or yellow mucus with or without a fever. | Call my contact person or doctor.<br>Start my treatment as soon as possible.<br>Start my antibiotic ____________ for ____ days.<br>____________________<br>____________________<br>Start Prednisone ____________ for ____ days.<br>____________________<br>**IF MY SYMPTOMS DO NOT IMPROVE AFTER 48 HOURS, I WILL CALL MY DOCTOR. IF IT IS AFTER OFFICE HOURS, I WILL GO TO THE EMERGENCY ROOM.** |

| I FEEL I AM IN DANGER | WHAT SHOULD I DO? |
|---|---|
| • I am extremely short of breath.<br>• I am confused, agitated or drowsy.<br>• I have sudden chest pain. | **CALL 911** |

## A respiratory educator can help you to manage your flare-up with an action plan.

It is important to see your doctor on a **regular basis** in order to help manage your COPD.

You should go to emergency when your breathing is getting much worse, your treatment is not improving your breathing or you feel uncomfortable staying at home because of your breathing.

If you do not see your doctor on a regular basis and instead go to emergency for ongoing care, you may not benefit from the follow-up that your doctor and health team can provide.

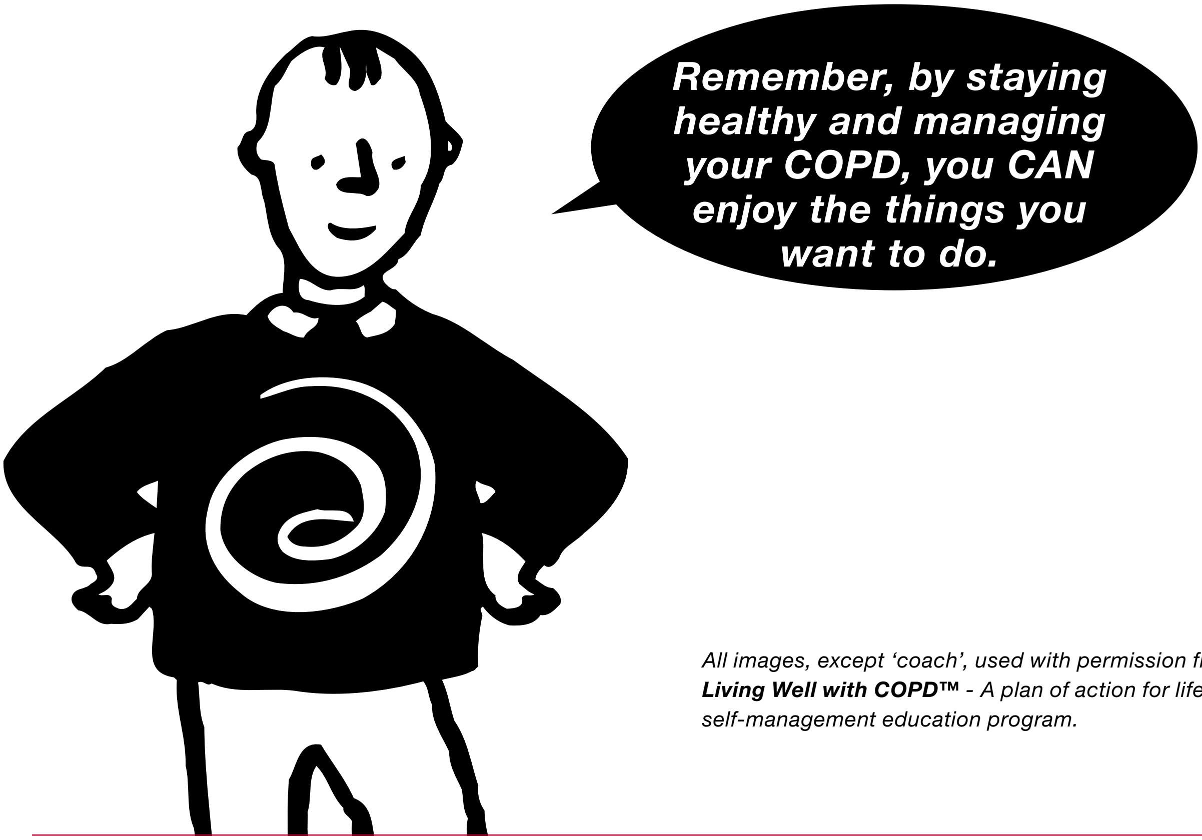


*All images, except 'coach', used with permission from **Living Well with COPD™** - A plan of action for life self-management education program.*

**Get the information and support you need from one of our Breathworks COPD educators.**

**Phone 1-866-717-COPD (2673) or visit us online at www.lung.ca/breathworks.**

BREATHWORKS™
