# Supplementary material for: Effectiveness of a pharmacist-driven intervention in COPD (EPIC): study protocol for a randomized controlled trial
Source: Trials. 2016 Oct 13;17:502. doi: 10.1186/s13063-016-1623-7 (PMC5064938; doi:10.1186/s13063-016-1623-7)
Supplement: Additional file 7: — COPD Action Plan. An action plan to be developed by the pharmacists in the intervention arm, in conjunction with the patient and their primary prescriber as needed, to provide the patients with direction in the event of an acute exacerbation. This document was developed by the Canadian Thoracic Society and Lung Association. (PDF 405 kb) [file 13063_2016_1623_MOESM7_ESM.pdf]

My COPD Action Plan ______________________ Date ____________

Patient's Copy (Patient's Name)

Canadian Respiratory Guidelines

COPD Treatable. Preventable.

This is to tell me how I will take care of myself when I have a COPD flare-up.

My goals are ____

____

My support contacts are ______________________ and ______________________
(Name & Phone Number) (Name & Phone Number)

My Symptoms	I Feel Well	I Feel Worse	I Feel Much Worse URGENT
I have sputum.	My usual sputum colour is: ______________	Changes in my sputum, for **at least** 2 days. *Yes* ☐ *No* ☐	My symptoms are not better after taking my flare-up medicine for 48 hours.
		**OR**	
I feel short of breath.	When I do this: ______________ ______________	More short of breath than usual for **at least** 2 days. *Yes* ☐ *No* ☐	I am very short of breath, nervous, confused and/or drowsy, and/or I have chest pain.

My Actions	Stay Well	Take Action	Call For Help
	I use my daily puffers as directed.	If I checked 'Yes' to one or both of the above, I use my **prescriptions** for COPD flare-ups.	I will call my support contact and/or see my doctor and/or go to the nearest emergency department.
	If I am on oxygen, I use _______L/min.	I use my daily puffers as usual. If I am **more** short of breath than usual, I will take ____ puffs of ___________ up to a **maximum** of ____ times per day.	**I will dial 911.**
		I use my breathing and relaxation methods as taught to me. I pace myself to save energy.	**Important information:** I will tell my doctor, respiratory educator, or case manager **within 2 days** if I had to use any of my flare-up prescriptions. I will also make follow-up appointments to review my COPD Action Plan twice a year.
		If I am on oxygen, I will increase it from ___ L/min to ___ L/min.	

Notes:

THE LUNG ASSOCIATION™ L'ASSOCIATION PULMONAIRE

CANADIAN THORACIC SOCIETY
SOCIÉTÉ CANADIENNE DE THORACOLOGIE

Produced in collaboration with the *COPD & Asthma Network of Alberta (CANA)*. *The Canadian Thoracic Society (CTS)* acknowledges the past contributions of *Living well with COPD* and the *Family Physician Airways Group of Canada*.

Canadian Respiratory Guidelines

COPD ACTION PLAN (Patient's copy)

Why do I need this COPD Action Plan?

- Your Action Plan is a written contract between you and your health care team. It will tell you how to manage your COPD flare-ups. Use it along with any other information you get from your health care team about managing your COPD every day.
- Your Action Plan will help you and your caregivers to quickly recognize and act to treat your flare-ups. This will keep your lungs and you as healthy as possible.

How will I know that I am having a COPD "flare-up"?

- You will often see a change in your amount or colour of sputum and/or you may find that you are more short of breath than usual. Other symptoms can include coughing and wheezing more.
- Your flare-up Action Plan is to be used only for COPD flare-ups. Remember that there are other reasons you may get short of breath, such as when you have pneumonia, are anxious, or have heart problems.
- Before or during a flare-up you may notice changes in your mood, such as feeling down or anxious. Some people have low energy or feel tired before and during a COPD flare-up.

What triggers a "COPD flare-up"?

- A COPD flare-up can sometimes happen after you get a cold or flu, or when you are stressed and run down.
- Being exposed to air pollution and changes in the weather can also cause COPD flare-ups. To learn about the daily air quality in your area, visit Environment Canada's Air Quality Health Index (AQHI) website at www.ec.gc.ca/cas-aqhi/ and click on 'Your Local AQHI Conditions'. Ask your health care team about ways to avoid all possible triggers.

When should I use this COPD Action Plan?

- Your COPD Action Plan is used only for COPD flare-ups.
- Remember that there are other reasons you may get short of breath, such as when you have pneumonia, are anxious, or have heart problems. If you become more short of breath but don't have symptoms of COPD flare-up, see a doctor as soon as possible.

REMEMBER:

- Learn about your COPD from a respiratory educator, credible websites, such as www.lung.ca, and education programs.
- Take your regular daily medicine as prescribed.
- Don't wait more than 48 hours after the start of a COPD flare-up to take your antibiotic and/or prednisone medicines. See your pharmacist quickly to get your prescriptions for COPD flare-up.
- When you start an antibiotic, make sure that you finish the entire treatment.
- Quitting smoking and making sure that your vaccinations are up-to-date (for flu every year and for pneumonia at least once) will help prevent flare-ups.
- Be as active as possible. Inactivity leads to weakness, which may cause more flare-ups or flare-ups that are worse than usual. Ask your doctor about pulmonary rehabilitation and strategies to help reduce your shortness of breath and improve your quality of life.
- Follow up with your doctor within 2 days after using any of your prescriptions for a COPD flare-up.

MY NOTES AND QUESTIONS:

My COPD Action Plan ________________________________ Date ______________
Patient's Copy (Patient's Name)

Canadian Respiratory Guidelines

COPD Treatable. Preventable.

This is to tell me how I will take care of myself when I have a COPD flare-up.

My goals are ____

My support contacts are ______________________________ and ______________________________
(Name & Phone Number) (Name & Phone Number)

Prescriptions for COPD flare-up (Patient to take to pharmacist as needed for symptoms)

These prescriptions may be refilled two times each, as needed, for 1 year, to treat COPD flare-ups. Pharmacists may fax the doctor's office once any part of this prescription has been filled.

______________________ ______________________
Patient's Name Patient Identifier (e.g. DOB, PHN)

1. (A) If **the colour** of your sputum **CHANGES**, start antibiotic ____________________ Dose:__________ #pills:______
How often______________________ for #days:____

(B) If the first antibiotic was taken for a flare-up in the **last 3 months**, use this different antibiotic instead:
Start antibiotic ____________________ Dose:__________ #pills:______
How often______________________ for #days:____

AND / OR

2. If you are **MORE short of breath** than usual, start prednisone ______________ Dose:__________ #pills:______
How often:______________________ for #days:____

Once I start any of these medicines, **I will tell** my doctor, respiratory educator, or case manager within **2 days**.

______________________ ______________________ ______________________
Doctor's Name Doctor's Fax Doctor's Signature

______________________ ______________________
License Date

THE LUNG ASSOCIATION™ L'ASSOCIATION PULMONAIRE

CANADIAN THORACIC SOCIETY SOCIÉTÉ CANADIENNE DE THORACOLOGIE

Produced in collaboration with the *COPD & Asthma Network of Alberta (CANA)*. *The Canadian Thoracic Society (CTS)* acknowledges the past contributions of *Living well with COPD* and the *Family Physician Airways Group of Canada*.

Canadian Respiratory
Guidelines

COPD ACTION PLAN (Patient's copy)

Why do I need this COPD Action Plan?

- Your Action Plan is a written contract between you and your health care team. It will tell you how to manage your COPD flare-ups. Use it along with any other information you get from your health care team about managing your COPD every day.
- Your Action Plan will help you and your caregivers to quickly recognize and act to treat your flare-ups. This will keep your lungs and you as healthy as possible.

How will I know that I am having a COPD "flare-up"?

- You will often see a change in your amount or colour of sputum and/or you may find that you are more short of breath than usual. Other symptoms can include coughing and wheezing more.
- Your flare-up Action Plan is to be used only for COPD flare-ups. Remember that there are other reasons you may get short of breath, such as when you have pneumonia, are anxious, or have heart problems.
- Before or during a flare-up you may notice changes in your mood, such as feeling down or anxious. Some people have low energy or feel tired before and during a COPD flare-up.

What triggers a "COPD flare-up"?

- A COPD flare-up can sometimes happen after you get a cold or flu, or when you are stressed and run down.
- Being exposed to air pollution and changes in the weather can also cause COPD flare-ups. To learn about the daily air quality in your area, visit Environment Canada's Air Quality Health Index (AQHI) website at www.ec.gc.ca/cas-aqhi/ and click on 'Your Local AQHI Conditions'. Ask your health care team about ways to avoid all possible triggers.

When should I use this COPD Action Plan?

- Your COPD Action Plan is used only for COPD flare-ups.
- Remember that there are other reasons you may get short of breath, such as when you have pneumonia, are anxious, or have heart problems. If you become more short of breath but don't have symptoms of COPD flare-up, see a doctor as soon as possible.

REMEMBER:

- Learn about your COPD from a respiratory educator, credible websites, such as www.lung.ca, and education programs.
- Take your regular daily medicine as prescribed.
- Don't wait more than 48 hours after the start of a COPD flare-up to take your antibiotic and/or prednisone medicines. See your pharmacist quickly to get your prescriptions for COPD flare-up.
- When you start an antibiotic, make sure that you finish the entire treatment.
- Quitting smoking and making sure that your vaccinations are up-to-date (for flu every year and for pneumonia at least once) will help prevent flare-ups.
- Be as active as possible. Inactivity leads to weakness, which may cause more flare-ups or flare-ups that are worse than usual. Ask your doctor about pulmonary rehabilitation and strategies to help reduce your shortness of breath and improve your quality of life.
- Follow up with your doctor within 2 days after using any of your prescriptions for a COPD flare-up.

MY NOTES AND QUESTIONS:

